# Structural and biochemical elucidation of class I hybrid cluster protein natively extracted from a marine methanogenic archaeon

**DOI:** 10.3389/fmicb.2023.1179204

**Published:** 2023-05-11

**Authors:** Olivier N. Lemaire, Mélissa Belhamri, Tristan Wagner

**Affiliations:** Max Planck Institute for Marine Microbiology, Bremen, Germany

**Keywords:** methanogenic archaea (MA), hybrid cluster protein, anaerobic biochemistry, structural biology, metalloenzyme active site, internal channel, nitric oxide

## Abstract

Whilst widespread in the microbial world, the hybrid cluster protein (HCP) has been paradoxically a long-time riddle for microbiologists. During three decades, numerous studies on a few model organisms unravelled its structure and dissected its metal-containing catalyst, but the physiological function of the enzyme remained elusive. Recent studies on bacteria point towards a nitric oxide reductase activity involved in resistance during nitrate and nitrite reduction as well as host infection. In this study, we isolated and characterised a naturally highly produced HCP class I from a marine methanogenic archaeon grown on ammonia. The crystal structures of the enzyme in a reduced and partially oxidised state, obtained at a resolution of 1.45 and 1.36-Å, respectively, offered a precise picture of the archaeal enzyme intimacy. There are striking similarities with the well-studied enzymes from *Desulfovibrio* species regarding sequence, kinetic parameters, structure, catalyst conformations, and internal channelling systems. The close phylogenetic relationship between the enzymes from *Methanococcales* and many *Bacteria* corroborates this similarity. Indeed, *Methanococcales* HCPs are closer to these bacterial homologues than to any other archaeal enzymes. The relatively high constitutive production of HCP in *M. thermolithotrophicus*, in the absence of a notable nitric oxide source, questions the physiological function of the enzyme in these ancient anaerobes.

## 1. Introduction

Hybrid clusters are metallo-cofactors constituted of a mixture of Fe atoms bridged with sulphur and oxygen atoms. The most notorious enzymes harbouring them as a prosthetic group are the hybrid cluster proteins (HCPs, also historically named prismane protein) (Aragão et al., 2008; Hagen, 2022), which are present in strict or facultative anaerobic archaea and bacteria [e.g., the bacterium *Desulfovibrio vulgaris* (Cooper et al., 2000) and the archaeon *Pyrococcus furiosus* (Overeijnder et al., 2009)], and even a few unicellular eukaryotes [e.g., the microalga *Chlamydomonas reinhardtii* (van Lis et al., 2020)]. Since the first publication in 1989 (Hagen et al., 1989), HCPs have been extensively studied by metal content determination (Moura et al., 1992; Pierik et al., 1992), different types of spectroscopy (Hagen et al., 1989; Moura et al., 1992; Pierik et al., 1992; Stokkermans et al., 1992; van den Berg et al., 1994; Marritt et al., 1995; Hagen et al., 1998; Kröckel et al., 1998; Tavares et al., 1998; Pereira et al., 1999; van den Berg et al., 2000; Macedo et al., 2002), redox titration (Pierik et al., 1992; Stokkermans et al., 1992; van den Berg et al., 1994, 2000), and X-ray crystallography (Cooper et al., 2000; Macedo et al., 2002, 2003; Aragão et al., 2003, 2008; Fujishiro et al., 2021), characterising the hybrid cluster in its different redox states. The cluster undergoes a complex chemistry with four oxidation states (reduced, semi-oxidised, oxidised, and super-oxidised, see Hagen, 2022) with distinct spectroscopic properties and structures also recently observed in the carbon monoxide dehydrogenase (CODH) type V. The CODH type V is homologous to HCP and shares the cluster chemical composition and a similar overall fold whilst increasing in complexity (Jeoung et al., 2022). The most studied HCPs are those found in *Desulfovibrio vulgaris* and *Desulfovibrio desulfuricans* (named below *Dv*HCP and *Dd*HCP, respectively), followed by the isoform found in *Escherichia coli* (*Ec*HCP). The data gathered on enzymes from other organisms remain scarce.

Despite the undertaken efforts, the physiological function of HCPs is still elusive 30 years after their discovery. Various studies showed that the protein is produced in the presence of nitrate/nitrite (van den Berg et al., 2000; Kim et al., 2003; Haveman et al., 2004; Beliaev et al., 2005; Filenko et al., 2005; He et al., 2006; Cadby et al., 2017), sulphate (Aragão et al., 2003), oxidative stress (Briolat and Reysset, 2002; Almeida et al., 2006), or during pathogenic infection (Okinaka et al., 2002; Kim et al., 2003; Roos and Klemm, 2006). HCP was initially shown to reduce hydroxylamine (a side product of nitrate/nitrite reduction) to ammonia, which would allow its assimilation (Wolfe et al., 2002). Hydroxylamine reduction was confirmed in different HCPs (Wolfe et al., 2002; Aragão et al., 2003; Cabello et al., 2004; Overeijnder et al., 2009), and the protein was shown to enhance cellular resistance to nitrite and hydroxylamine (Cabello et al., 2004; Yurkiw et al., 2012), allowing nitrogen acquisition (Cabello et al., 2004). Nevertheless, the kinetic parameters of the enzyme (*K*_M_ in the millimolar range, low activity compared to other enzymes, especially at physiological pH) are incompatible with a physiological hydroxylamine reductase function (Almeida et al., 2006; Cole, 2021; Hagen, 2022). The activity is considered a side reaction, as it has been shown for other enzymes, including the Ni-dependent CODH homologous to HCPs (Mager, 1960; Faeder et al., 1974; Ostrowski et al., 1989; Heo et al., 2002; Hagen, 2022; Jeoung et al., 2022). A peroxidase function was later demonstrated (Almeida et al., 2006), but the gene expression was not systematically correlated during oxidative stress (Zhang et al., 2006; Wang et al., 2022), and the kinetic parameters (catalytic turnover ranging from 0.05 to 0.17 s^−1^, concomitant production of more efficient peroxidases in the culture conditions) again appeared incoherent with a physiological peroxidase function (Cole, 2021; Hagen, 2022). More recently, the HCP was shown to act as high-affinity nitric oxide (NO)-reductase, detoxifying NO into nitrous oxide N_2_O at physiological concentrations, and its overexpression protects cells against nitrosative stress (Kim et al., 2003; Wang et al., 2016; Hagen, 2019). Accordingly, HCP production depends on a NO-inducible promotor in some organisms, in agreement with a possible role in the nitrate/nitrite reduction pathway as the nitrite reduction is a source of NO (Filenko et al., 2007; Spiro, 2007; Wang et al., 2016; Bulot et al., 2019; Gao et al., 2019; Yu et al., 2020). The similar effects/co-occurrence of reactive oxygen species, NO/nitrosylative stress, and the promiscuity of NO for oxidative stress sensors would explain the production of HCP during oxidative stress (Filenko et al., 2007). The NO reductase activity was later amended by a role in protein transnitrosylation (Seth et al., 2018) in which HCP generates R-S-nitrosothiol from NO. If the exact process of HCP-dependent protein nitrosylation has not been fully unveiled yet [and is still debated (Cole, 2021; Hagen, 2022)], spectroscopic analysis confirmed the existence of a dinitrosyl form of the hybrid cluster, thought to be an intermediate during NO reduction to N_2_O, that could represent a nitrosyl donor for nitrosylation (Hagen, 2019). Finally, an HCP study on a photosynthetic microalga speculates a nitrate-reducing S-nitrosylase function that has to be experimentally proven (van Lis et al., 2020). The HCP physiological function appears to be on the path to being solved. Yet, alternative functions might exist depending on the organism, the growth conditions, or the HCP class/isoform.

Three HCP classes were historically sorted based on their sequences (van den Berg et al., 2000; Hagen, 2022). Classes I and II differ by a 4-residue insertion in the cubane [4Fe-4S] cluster binding motif, whilst class III exhibits a reduced N-terminal domain. Recent phylogenetic analysis pointed out that these classes do not form phylogenetic groups: if class III forms a branch distinct from both other classes, class II appears to be a subgroup of class I (van Lis et al., 2020). The same study suggested that class III even precedes the separation of the life domains and would be present in the last universal common ancestor (LUCA) (van Lis et al., 2020; Hagen, 2022; Jeoung et al., 2022). The gene coding for class II HCP, such as EcHCP, is often found in the genome next to a gene coding for the HCP reductase, a NAD(P)H oxidising flavoenzyme involved in oxidoreduction of the HCP and whom equivalent in class I and III is not known (van den Berg et al., 2000; Hagen, 2022). Another noticeable difference between HCP classes is that HCP class III appears to be dimeric, whilst all characterised classes I and II were shown to be monomeric [with the exception of one report in the case of nitrosylated enzyme (Seth et al., 2018)].

Many archaeal genomes harbour a gene coding for a putative HCP belonging exclusively to class I or III (van Lis et al., 2020). The sole archaeal HCP biochemically studied is from *Pyrococcus furiosus* and belongs to class III (Overeijnder et al., 2009). Noteworthy, the only available structure of an archaeal HCP is from the thermophilic methanogen *Methanothermobacter marburgensis*. According to our knowledge, this class III representative [available in the PDB database under ID 7E0L (Fujishiro and Takaoka, 2022)] has yet to be described in any publication. This structure will not be used for more than sequences and comparisons to respect the authors’ study.

The presence of HCP in methanogens is unexpected as most of these ancient organisms are not known to use nitrate, nitrite, or NO as these oxidants are toxic to them (Klüber and Conrad, 1998). Recent transcriptomics studies on the nitrogen metabolism from the thermophilic archaeon *Methanothermococcus thermolithotrophicus* have been investigated by our group and showed a constitutively rather high expression of an HCP class I at a level comparable to that of genes coding for enzymes involved in methanogenesis (Maslać et al., 2022). In this study, we purified the HCP from *M. thermolithotrophicus* grown on ammonia as the only nitrogen source and obtained its structure in reduced and semi-oxidised states. The conservation in architecture, active site, reactions, and internal tunnelling systems is discussed. Phylogenetic analyses show that the HCPs from *Methanococcales* species are closer to bacterial HCPs than to any archaeal enzymes, which further questions their physiological functions in methanogens.

## 2. Materials and methods

### 2.1. Strains and growth conditions

*Methanothermococcus thermolithotrophicus* strain DSM 2095 was obtained from the Deutsche Sammlung von Mikroorganismen und Zellkulturen (DSMZ, Braunschweig, Germany). The archaeon was grown on ammonia and sulphate in a fermenter at 65°C, as described in Jespersen et al. (2023).

### 2.2. Protein purification

The purification of the native *Mt*HCP was originally performed based on its colour and the natural abundance of the protein. It was purified several times with a similar, reproducible protocol. The optimal one is described below. The heterologous production of the enzyme was not attempted.

Cell lysis was performed under an N_2_/CO_2_ (90:10%) atmosphere. About 19 g (wet weight) of frozen cells were thawed and diluted with four volumes of lysis buffer (50 mM tricine/NaOH pH 8.0, 2 mM dithiothreitol (DTT)), sonicated (3 × 10 s at 70% power, probe KE76 Bandelin SONOPULS Berlin, Germany), and centrifuged for 1 h at 45,000 x *g* at 18°C. The following overall procedure was performed under yellow light and at 20°C. The supernatant was transferred to an anaerobic Coy tent containing an N_2_/H_2_ (97:3%) atmosphere, filtered through a 0.2 μm filter (Sartorius, Göttingen, Germany), and loaded on a 20 ml HiTrap DEAE-Sepharose fast flow column (GE Healthcare, Munich, Germany). Elution was performed with a 100–600 mM NaCl linear gradient for 15 column volumes (CVs) at a flow rate of 2.5 ml/min. The sample of interest eluted between 125 and 310 mM NaCl. The resulting pool was diluted with four volumes of lysis buffer, filtered, and loaded on a 15 ml HiTrap Q-Sepharose High-Performance column (GE Healthcare, Munich, Germany). Elution was performed with a linear gradient ranging from 0 to 500 mM NaCl for 13.33 CV at a flow rate of 2 ml/min. The fractions of interest was eluted between 248 and 320 mM NaCl. The resulting pooled fraction was diluted with four volumes of 25 mM Tris/HCl) pH 7.6, 2 M (NH_4_)_2_SO_4_, and 2 mM DTT, filtered through a 0.2 μm filter (Sartorius, Göttingen, Germany), and injected on a 5 ml HiTrap Phenyl Sepharose High-Performance column (GE Healthcare, Munich, Germany). The sample was eluted with a 1.8 to 0 M linear gradient of (NH_4_)_2_SO_4_ for 24 CV at a flow rate of 1.5 ml/min. The protein was eluted between 0.41 and 0.25 M (NH_4_)_2_SO_4_. After concentration up to 400 μl with a 10-kDa cutoff centrifugation filter (Merck, Darmstadt, Germany), contaminants were separated by size-exclusion chromatography on a Superose 6 Increase 10/300 GL (GE Healthcare, Munich, Germany) in 25 mM Tris/HCl pH 7.6, 2 mM DTT, and 10% (v/v) glycerol at a flow rate of 0.4 ml/min. The final pool was concentrated with a 10-kDa cutoff centrifugation filter (Merck, Darmstadt, Germany), and protein concentration was estimated by the Bradford method (Bio-Rad Laboratories, Munich, Germany). Each purification step was systematically controlled by denaturing sodium dodecyl sulphate polyacrylamide gel electrophoresis (SDS-PAGE). The protein was directly used for crystallisation or aliquoted and stored frozen at −80°C.

### 2.3. Protein crystallisation

Crystallisation was performed anaerobically by initial screening at 20°C using the sitting drop method on 96-Well MRC 2-Drop polystyrene Crystallisation Plates (SWISSCI) in a Coy tent containing an N_2_/H_2_ (97:3%) atmosphere. The reservoir chamber was filled with 90 μl of crystallisation condition (JBScreen Wizard crystallisation screen, Jena Bioscience), and the crystallisation drop was formed by spotting 0.55 μl of purified protein with 0.55 μl of precipitant. Both structures were obtained from the same drop, opened several months apart. The protein was crystallised at 9.9 mg/ml in a solution containing 20% (w/v) PEG 3,350 and 200 mM magnesium formate. Densities in the electron density map suggest contamination from another crystallisation condition spatially close and containing 30% (v/v) 2-methyl-2,4-pentanediol, 20 mM calcium chloride, and 100 mM sodium acetate, pH 4.6.

For the reduced state, the crystals were soaked in the crystallisation solution supplemented with 20% (v/v) glycerol for a few seconds before freezing in liquid nitrogen. For the reduced/oxidised state, the crystals were soaked in the crystallisation solution supplemented by 100 mM hydroxylamine/HCl for 5.7 min and then transferred in the crystallisation solution supplemented with 20% (v/v) ethylene glycol for a few seconds before freezing in liquid nitrogen.

### 2.4. Data collection and structural analysis

The diffraction experiments were performed at 100 K on the beamline P11 from DESY and PXIII (X06DA) from the Swiss Light Source (SLS). The data were processed and scaled with *autoPROC* (Vonrhein et al., 2011). The data of the structure of *Mt*HCP in the reduced/oxidised state presented anisotropy (along the following axes: *a* = 1.451 Å, *b* = 1.343 Å, and *c* = 1.748 Å) and was further processed with *STARANISO* correction integrated with the *autoPROC* pipeline (Tickle et al., 2018) (STARANISO. Cambridge, United Kingdom: Global Phasing Ltd.).

The structure of the reduced *Mt*HCP was solved by performing a single anomalous dispersion experiment at the Fe K-edge (Supplementary Table S1) using the *SHELX* package (Usón and Sheldrick, 2018). The first model was then refined by extending the resolution to 1.45 Å by a second dataset. The reduced state was used as a template to solve the reduced/oxidised state structure by molecular replacement with *PHASER* from the *PHENIX* package (Liebschner et al., 2019). The resolution limit was determined based on the obtained electron density map quality and Rpim as major criteria. All models were manually built *via* COOT (Emsley et al., 2010) and refined with *PHENIX* (version 1.20.1-4487). The last refinement steps were performed by refining with translation libration screw (TLS) for the reduced state and considering all atoms anisotropic for the reduced/oxidised state and were validated by the MolProbity server (Chen et al., 2010)^1^. Both models were refined with hydrogens in the riding position. Hydrogens were omitted in the final deposited models. The PDB ID codes of the structures are 8CNR and 8CNS for the *Mt*HCP_red_ and *Mt*HCP_mix_ structures, respectively. Data collection and refinement statistics for the deposited models are listed in Supplementary Table S1. All figures were generated and rendered with PyMOL (Version 2.2.0, Schrödinger, LLC, New York, NY, United States). The structural superpositions in Supplementary Table S2 and Supplementary Figure S3 were performed with PyMOL or the secondary-structure matching (SSM) tool from the CCP4 suite (Krissinel and Henrick, 2004; Winn et al., 2011). For the elaboration of Supplementary Figure S3, the B-factor column of 8CNR was replaced by the residues deviation obtained from SSM by the PyMOL script data2bfactor.py written by Robert L. Campbell. The protein was then coloured by residues deviation. Non-aligned residues, residues with aberrant deviation, and fragments of three residues or less were omitted for clarity. Internal tunnel predictions were performed by the *CAVER* tool (Kozlikova et al., 2014) by applying a probe radius of 1.0 Å and starting from the Cβ of the cysteine at position 455 (*Mt*HCP) or the equivalent cysteine.

### 2.5. High-resolution clear native PAGE (hrCN PAGE) and size-exclusion chromatography

The hrCN PAGE protocol was adapted from Lemaire et al. (2018). Glycerol was added to the sample at a final amount of 20% (v/v). Ponceau S at a final concentration of 0.001% (w/v) served as a marker to follow the migration. The buffer composition for the electrophoresis cathode was the following: 50 mM tricine, 15 mM Bis-Tris/HCl, pH 7.0, 0.05% (w/v) sodium deoxycholate, 2 mM DTT, and 0.01% (w/v) dodecyl maltoside, whilst the anode buffer contained 50 mM Bis-Tris/HCl, PH 7.0, 2 mM DTT. An 8–15% linear polyacrylamide gradient gel was used, and electrophoresis was run under a N_2_/CO_2_ (90:10%) atmosphere with a constant 40 mA current (PowerPac^™^ Basic Power Supply, Bio-Rad). After electrophoresis, protein bands were visualised with Ready Blue^™^ Protein Gel stain (Sigma Aldrich, Hamburg, Germany). The native protein ladder used is NativeMark^™^ Unstained Protein Standard (Thermo Fischer Scientific, Driesch, Germany).

The determination of the oligomeric state by gel filtration was performed in triplicate on a Superose 6 Increase 10/300 GL (GE Healthcare, Munich, Germany) in 25 mM Tris/HCl pH 7.6, 2 mM DTT, 10% (v/v) glycerol at a flow rate of 0.4 ml/min, and in an anaerobic Coy tent containing an N_2_/H_2_ (97:3%) atmosphere. High molecular weight range gel filtration calibration kit (GE Healthcare, Munich, Germany) was used as the protein standard.

### 2.6. Absorbance spectra

The protein absorbance spectra were monitored aerobically and anaerobically. The aerobic oxidised spectrum was monitored using a Cary 60 UV–Vis spectrophotometer (Agilent Technologies). Absorbance spectra were measured by scanning the wavelength from 250 to 600 nm, with a measurement every 0.5 nm, in a 1 mm-path length TrayCell (Hellma Analytics) at room temperature. The protein concentration was 26.4 mg/ml, and the baseline absorbance spectrum of the buffer was subtracted from the measured protein spectrum. The anaerobic spectra were monitored on a BMG Labtech FLUOstar Omega Microplate reader in an anaerobic chamber filled with an N_2_ (100%) atmosphere at room temperature. The protein concentration was 3.4 mg/ml, and the baseline absorbance spectrum of the buffer was subtracted from the measured protein spectrum. The spectrum was monitored on a 384 wells transparent plate using an 18 μl sample volume. The protein was reduced or oxidised by the addition of 200 μM of sodium dithionite or potassium ferricyanide, respectively.

### 2.7. Enzymatic assays

The hydroxylamine reductase assay was performed according to the protocol set up by Wolfe et al. (2002). The activity was monitored by the enzyme-dependent decrease in the absorbance of methyl viologen (MV) at 600 nm on a BMG Labtech FLUOstar Omega Microplate reader in an anaerobic chamber filled with an N_2_ (100%) atmosphere. For practical reasons (physical limits of the equipment and limitation of the evaporation), activities were monitored at 50°C. Measurements were acquired in 100 mM CHES/NaOH pH 9.0 or in 50 mM HEPES/HCl pH 7.35 with the addition of 10 μM EDTA, 10 mM MV, and 75 μM sodium dithionite. The final protein concentration was 26.4 μg/ml at pH 7.35 and 2.64 µg/ml at pH 9.0 (200 µl final volume), and the tested hydroxylamine concentration ranged from 0 to 105 mM final. At pH 9.0, activities with a hydroxylamine concentration above 15 mM could not be reliably measured as it appears to induce reproducible drops in enzyme activity, probably due to the combined high temperature and pH. Enzyme-independent viologen oxidation after hydroxylamine addition, proportional to hydroxylamine concentration, had to be subtracted for every measurement. The molar extinction coefficient of MV used for calculation was determined experimentally in our working conditions (12,791 M^−1^. cm^−1^ and 13,489 M^−1^. cm^−1^ at pH 7.35 and 9.0, respectively). The activity measurements at both pHs can be fitted with a similar coefficient of determination using a Michaelis–Menten or a Hill equation, yet at pH 7.35, the Hill equation appears to be a better fit (*R*^2^ = 0.963 vs. 0.932 for the Michaelis–Menten equation). No cooperativity mechanism has been observed or proposed for other HCPs, and there is no experimental evidence for a reaction mechanism in HCP that would deviate from a Michaelis–Menten equation. The difference in *R*^2^ observed in the fits of our experiments is insufficient to question the proposed reaction mechanism in HCP, and the kinetic parameters used in the text were determined assuming a Michaelis–Menten equation. All measurements have been done in triplicates using a protein obtained from a unique purification and are presented in μmol of reduced hydroxylamine per minute per mg of protein, considering a mole of hydroxylamine reduced for two moles of viologen oxidised.

The peroxidase assay was performed aerobically according to the protocol set up by Almeida et al. (2006). The activity was monitored by following the enzyme-dependent decrease in the absorbance of sodium ascorbate at 290 nm (ε_290nm_ of 2,800 M^−1^.cm^−1^) due to the oxidation of ascorbate by hydrogen peroxidase reduction, using quartz cuvettes and a Cary 60 UV–Vis spectrophotometer (Agilent Technologies). The reaction mixture (400 μl) contained 50 mM potassium phosphate buffer, pH 7.0, and 0.2 mM sodium ascorbate. The experiment was assessed at 30, 40, 50, and 60°C. The used hydrogen peroxide concentration varied from 1 mM to 10 mM, and the *Mt*HCP concentration varied from 3.3 μg/ml to 132 μg/ml. Appropriate controls were performed for each assay and showed that no enzyme-dependent significant decrease in absorbance could be detected in any reproducible assay.

### 2.8. Sequence alignments and phylogenetic analysis

The protein sequence of *Mt*HCP was used as a query for BLAST (Altschul et al., 1997) in Protein Data Bank (PDB) and in the RefSeq database. The RefSeq database was selected to limit species redundancy and because it usually contains genomes of non-questionable quality. The search in the PDB extracted the sequences of structurally characterised HCPs (Cooper et al., 2000; Macedo et al., 2002; Aragão et al., 2003; Macedo et al., 2003; Fujishiro et al., 2021; Fujishiro and Takaoka, 2022). The maximal E-value (9 × 10^−122^) was with *Mm*HCP (PDB 7E0L). The searches in the RefSeq were set up to extract 500 sequences without additional parameters for the first research (maximal E-value 0.0) and limited to the *Archaea* kingdom for the second (maximal *E*-value for HCP, 1 × 10^−108^, *E*-value for CODH 1 × 10^−13^). The Supplementary Figure S2 was constructed using the sequence of MtHCP and the sequences extracted from the BLAST search in the PDB. It was designed with ESPript 3 (Robert and Gouet, 2014) by using an alignment constructed with Clustal Omega (Sievers et al., 2011).

The phylogenetic trees were constructed using the maximum likelihood method and were generated with the MEGA programme (Kumar et al., 2018) by using an alignment constructed with MUSCLE (Edgar, 2004). A total of 200 replicates were used to calculate each node score. The first presented tree was constructed with the sequences from the non-limited search in the RefSeq databank and the results from the PDB. The second tree was constructed with the sequences from the search in the RefSeq databank limited to *Archaea*, in which the sequences corresponding to CODH were manually deleted except one (CODHα from *Methanospirillum hungatei*, WP_011448292.1) and the results from the PDB. The tree shown in Supplementary Figure S11 was constructed with the sequences obtained from the three BLAST results (in the PDB and in the RefSeq database, non-restricted, and restricted to *Archaea* in which CODH sequences were deleted).

## 3. Results

### 3.1. *Mt*HCP overall architecture

**Figure 1:**
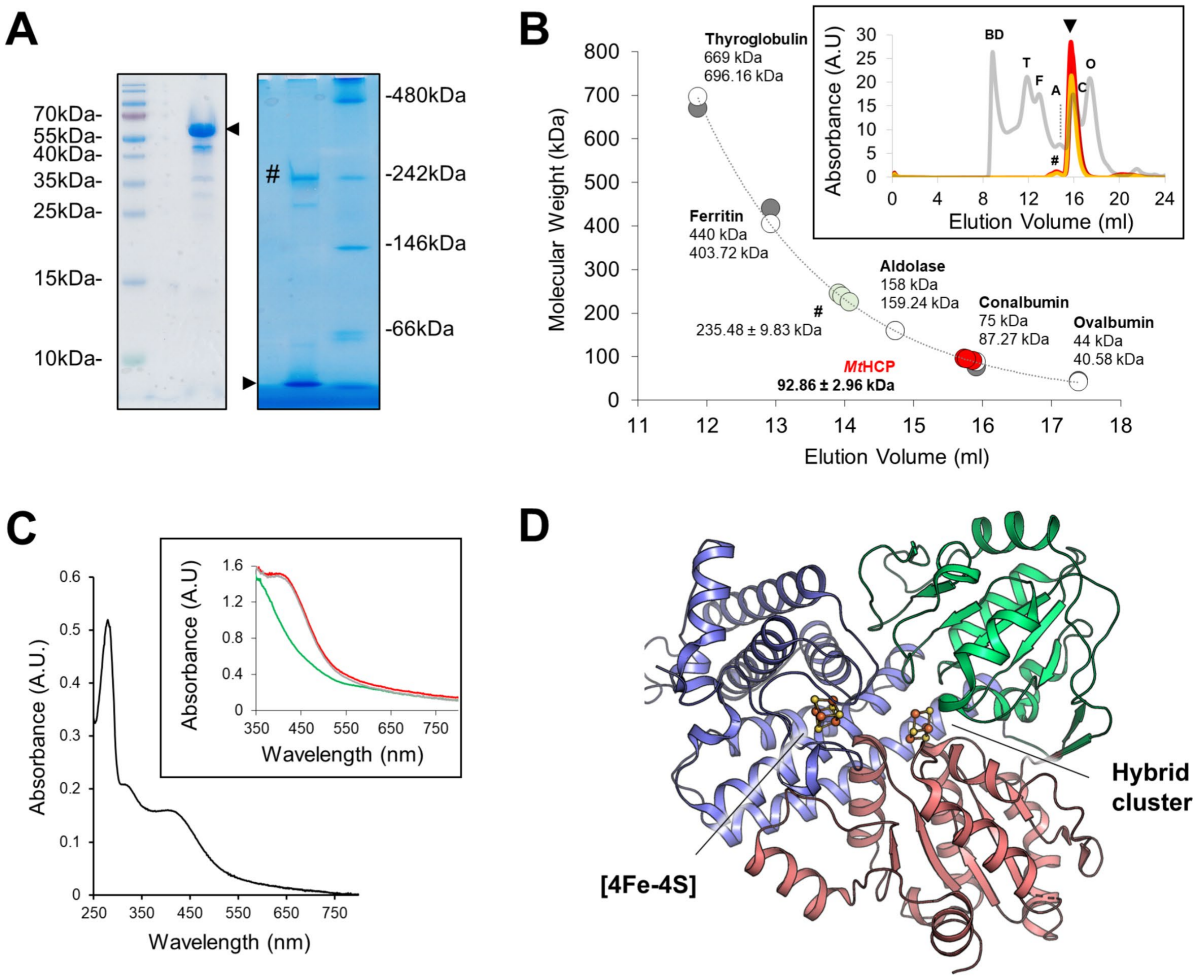
Oligomeric state, absorbance spectrum, and *Mt*HCP overall structure. **(A)** SDS-PAGE (left) and high-resolution Clear Native PAGE (right) profiles of the enriched *Mt*HCP fraction. About 3 μg of the sample kept frozen were loaded on gels. A black arrow indicates the position of *Mt*HCP, and a “#” symbol marks the main contaminant of *Mt*HCP. **(B)** Molecular weight estimation by size-exclusion chromatography. Protein standards (GE Healthcare, grey line in the insert) and three samples of *Mt*HCP (deep red, red, and orange lines in the insert) are overlaid. The measured elution volume of the standard is plotted (grey dots, upper sizes), and the extracted exponential curve (*y* = 310753e^−0.514x^, *R*^2^ = 0.9927) was used for molecular weight estimation. The theoretical size of the standard proteins (white dots, smaller sizes) is given for reference. BD stands for blue dextran, T for thyroglobulin, F for ferritin, A for aldolase, C for conalbumin, and O for ovalbumin. **(C)** Absorbance spectra of *Mt*HCP after freezing and storage at −80°C. The aerobic spectrum is coloured black in the main graph. Anaerobic measurements as isolated (red line), dithionite-reduced (green line), and ferricyanide-oxidised (grey line) are shown in the insert. **(D)** Overall structure of the *Mt*HCP. The protein is presented in cartoon coloured by domains (N-terminal, central, and C-terminal domains being coloured blue, green, and red, respectively) with the clusters presented as ball and stick. Sulphur and iron are coloured yellow and orange, respectively.

The native HCP from the thermophilic methanogen *M. thermolithotrophicus* (*Mt*HCP) was purified anaerobically by multiple chromatography steps and immediately crystallised without freezing. The purified brown protein fraction presents a major band of approximately 60 kDalton (Da) on SDS-PAGE (Figure 1A, *Mt*HCP expected molecular weight of 59,665.17 Da). The native electrophoresis and gel filtration mostly fit a monomeric organisation of the enzyme (Figures 1A,B). The spectrophotometric absorbance spectrum is typical of the [4Fe-4S] cluster-containing proteins and similar to previously published spectra of HCPs (Moura et al., 1992; Pierik et al., 1992; Stokkermans et al., 1992; Pereira et al., 1999; van den Berg et al., 2000; Figure 1C). Even if purified anaerobically and in the presence of dithiothreitol in the storage buffer, the spectrum of the frozen purified enzyme suggests an oxidised state that might be due to the long storage at −80°C (Figure 1C). The crystal structure was solved by single-wavelength anomalous dispersion at the Fe K-edge (SAD) and refined to a 1.45-Å resolution (see Materials and methods, Figure 1D, Supplementary Figures S1A,B, and Supplementary Table S1). *Mt*HCP is a monomeric 548-amino acid peptide organised in three domains (first domain residues 1–222, second domain 223–368, and third domain 369–548) harbouring a cubane [4Fe-4S] cluster and the hybrid cluster (Figure 1D). Based on the nomenclature, *Mt*HCP would belong to class I, coherently with cluster binding motif and its monomeric state confirmed in the crystal structure (Supplementary Table S2 and Supplementary Figure S2). Five extra residues, not expected in the available sequence (due to the incorrect prediction of the initiating methionine, WP_026182881.1), span from the N-terminal. The overall enzyme architecture is highly similar to the available HCP structures from bacteria (Supplementary Figure S3 and Supplementary Table S2). The major differences in structures are located on peripheral loops and helices. The well-defined electron density shows no sign of nitrosylation in the active site surroundings.

**Figure 2:**
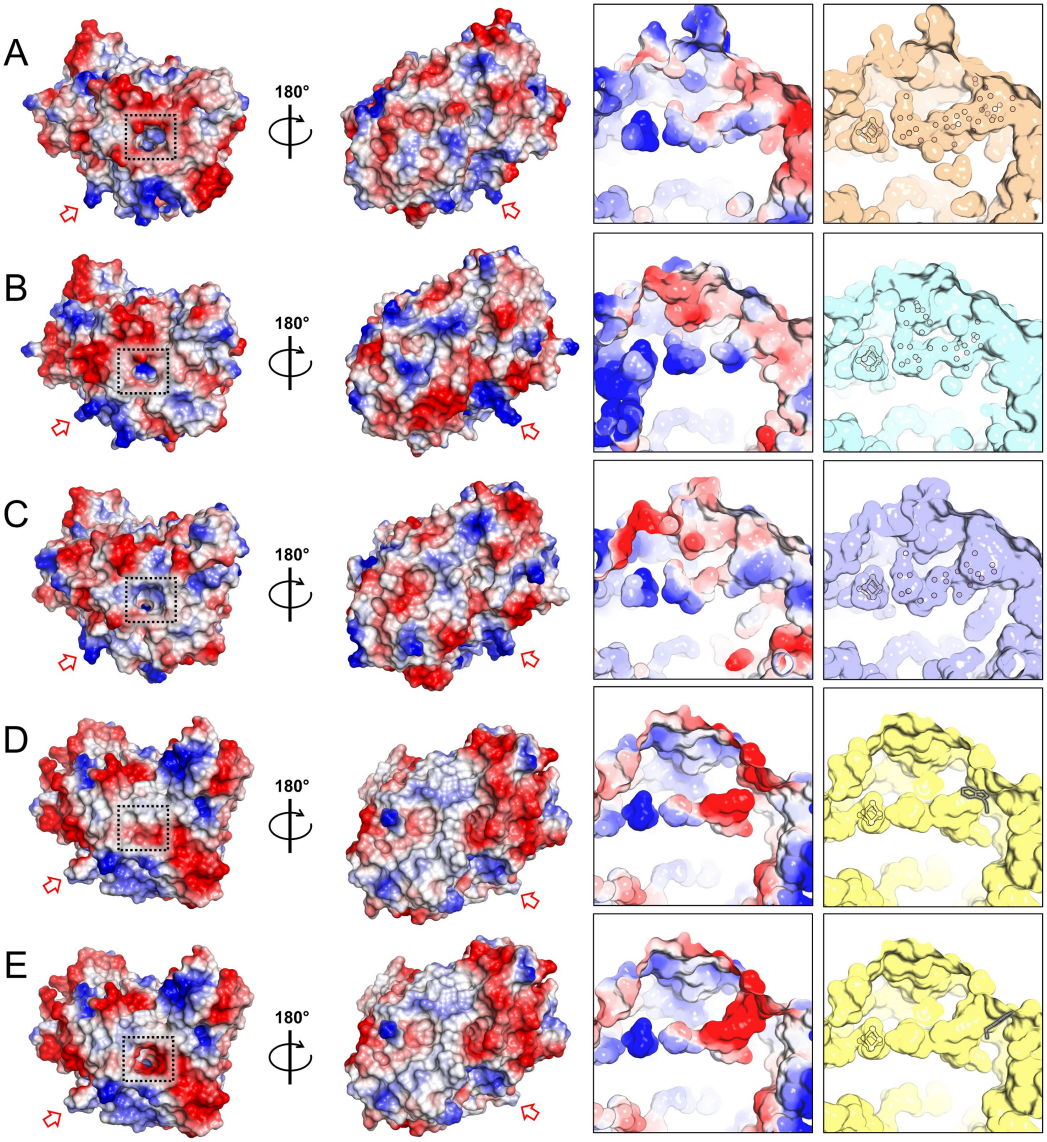
Charge distribution and access to the [4Fe-4S] cluster in HCP structures. **(A–E)** Charge distribution on the protein surface (left) and on the polar cavity near the cluster (right). Left: the proteins are shown in two orientations. A dashed black square highlights the entry of the polar cavities and the red arrow points to the loop proposed to interact with the partner in *Ec*HCP and its equivalent in other structures. Right panel: surface of the polar cavity and filled water molecules. The water molecules, [4Fe-4S] cluster, and tryptophan 193 (framed in grey) of *Ec*HCP are shown as balls and sticks. The structures presented are *Mt*HCP_red_ (PDB 8CNR, **A**), *Dd*HCP_red_ (PDB 1OA0, **B**), *Dv*HCP_red_ (PDB 1OA1, **C**), *Ec*HCP (PDB 7DE4, **D**), and the *Ec*HCP structure with a different conformation of tryptophan 193 **(E)**.

Differences in surface charges distribution were noticed between HCP class I for *Desulfovibrio* species and class II for *E. coli* (Fujishiro et al., 2021). An inspection of the surface charges in *Mt*HCP nuances the contrasts between classes I and II (Figure 2). *Mt*HCP exhibits a large negatively charged area on its surface surrounding a ~ 17-Å invagination towards the [4Fe-4S] cluster (Figure 2), also conserved in the bacterial structures. The cavity is polar, filled with water molecules in all structures, and exhibits a positively charged depth close to the cluster (Figure 2). It is interrupted in the structure of *Ec*HCP by the tryptophan at position 193 (Figure 2D) in a loop that is displaced in class I HCP structures. However, considering the loose electron density for this residue, the negatively charged cavity might be open to the solvent (modelled in Figure 2E). Furthermore, the residue is not conserved in all class II HCPs.

### 3.2. Structure of the active site in reduced and oxidised states

**Figure 3:**
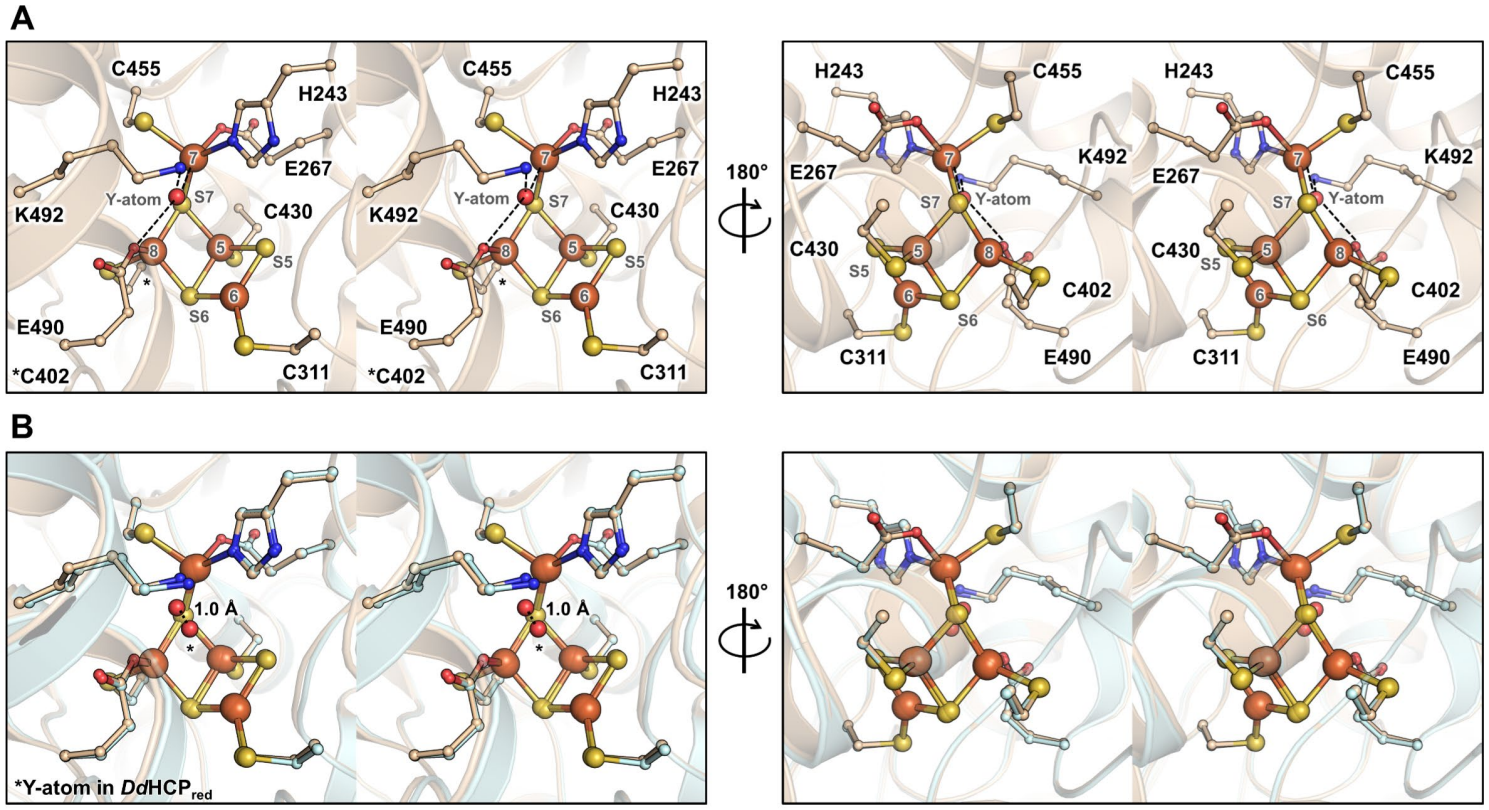
Overall organisation and active site of the isolated *Mt*HCP. **(A,B)** Stereo representation of the active site in two different orientations in the structures of *Mt*HCP_red_ **(A)** and superimposition of the structures of *Mt*HCP_red_ and *Dd*HCP_red_ (PDB 1OA0, **B**). The residues coordinating or interacting with the cluster are shown as balls and sticks. The structures are shown as transparent cartoons with the *Mt*HCP structure coloured wheat and the *Dv*HCP_red_ coloured light cyan. Oxygen, nitrogen, sulphur, and iron are coloured red, blue, yellow, and orange, respectively. The atom of the cluster is labelled according to previous studies (Hagen, 2022). The distance between the position of the Y-atom in *Mt*HCP_red_ and *Dd*HCP_red_ (modelled as oxygen in both structures) is shown as a dashed line and indicated in Ångström.

The structure harbours a hybrid cluster in a [4Fe-3S] composition, matching the bacterial homologues in their reduced state (Figure 3 and Supplementary Figure S1). The active sites of *Mt*HCP and the 1.25-Å resolution structure of the reduced *Dd*HCP are superimposable. Notably, residues surrounding the hybrid cluster are remarkably conserved between the sequences from *Desulfovibrio* species and *M. thermolithotrophicus* (Figure 3 and Supplementary Figure S2). The main difference is a 1.0-Å shift of the “Y-atom” at the hybrid cluster vicinity, which has also been attributed to an oxygen atom in *Mt*HCP based on the electron density (Figure 3). Because of the unequivocal chemical composition [4Fe 2μ3-S μ2-S (O)] of the hybrid cluster, we propose that the as-isolated *Mt*HCP represents the reduced state (Supplementary Figure S1C, the nomenclature *Mt*HCP_red_ will be used below). The difference between the reduced state in the structure and the oxidised state observed in spectrophotometry could come from the prolonged storage freezing at −80°C that might have led to the oxidation of the protein (Figure 1C). Alternatively, the crystallisation may have conserved or triggered the cluster reduction.

**Figure 4:**
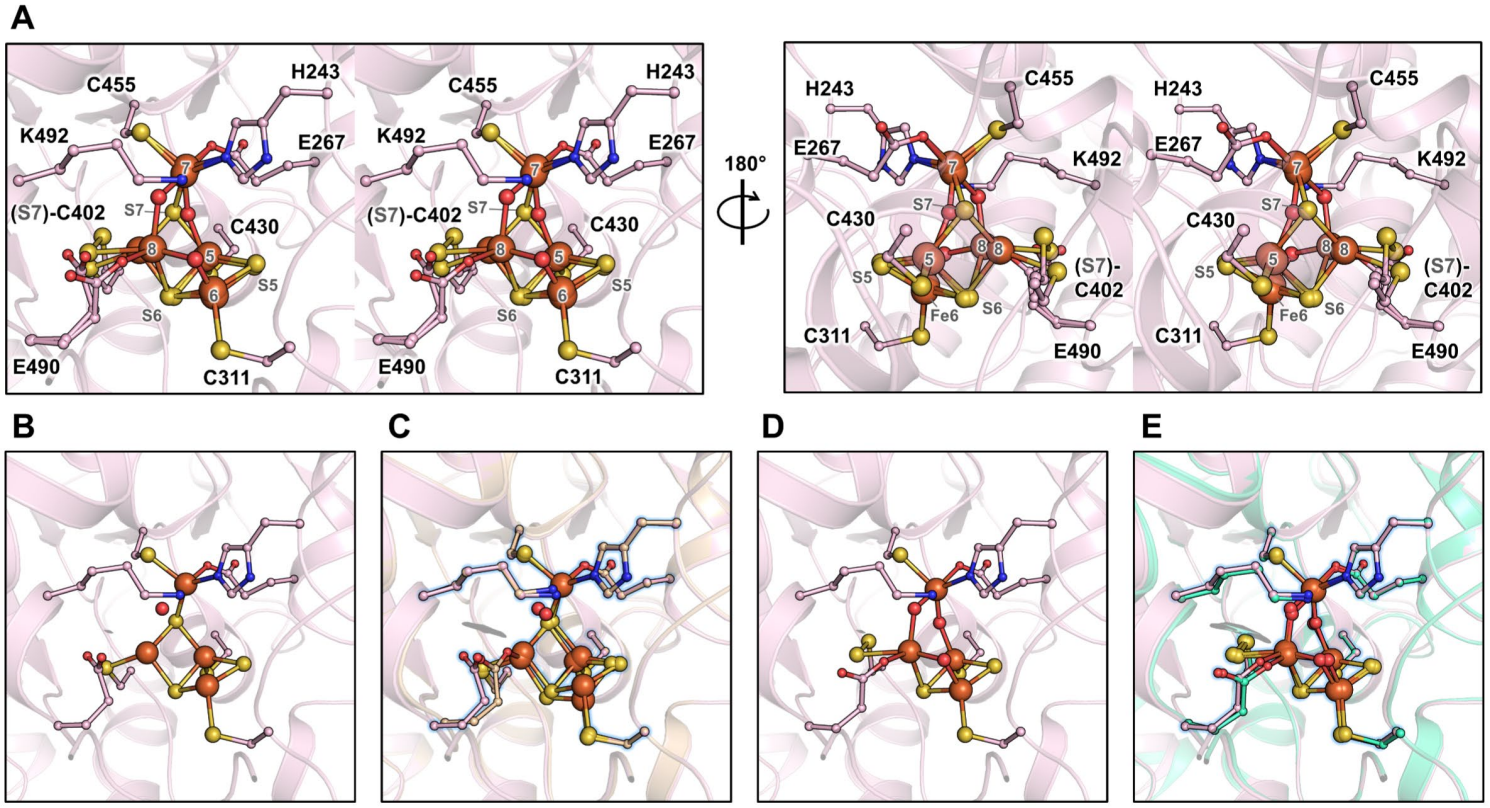
Active site of the *Mt*HCP in mixed redox state and the isolated states. **(A)** Stereo representation of the active site of the *Mt*HCP_mix_ containing a mixture of reduced and oxidised/semi-oxidised states. **(B)** Reduced state of the cluster extracted from the *Mt*HCP_mix_ structure. **(C)** Superimposition of the extracted structure of the reduced state and *Mt*HCP_red_. **(D)** Oxidised/semi-oxidised state (*Mt*HCP_ox_) of the cluster extracted from the *Mt*HCP_mix_ structure. **(E)** Superimposition of the *Mt*HCP_ox_ structure and the structure of the oxidised state of *Dd*HCP (PDB 1GNL). **(A–E)** The structures are shown as transparent cartoons, with the hybrid cluster represented as spheres and the coordinating/interacting residues as balls and sticks. *Mt*HCP_mix_ and its extracted structures are coloured light pink, the *Mt*HCP_red_ structure is coloured wheat, and the *Dd*HCP_ox_ structure is coloured green cyan. Oxygen, nitrogen, sulphur, and iron are coloured red, blue, yellow, and orange, respectively. **(C,E)** The extracted states of *Mt*HCP_mix_ are highlighted by a blue glow for clarity.

In order to obtain a different state of the active site or an intermediate of reaction, crystals were soaked in a solution of hydroxylamine (non-physiological substrate, 100 mM final) for a few minutes under anaerobic conditions before freezing in liquid nitrogen. The crystal structure was refined to 1.36-Å resolution (Supplementary Table S1), and the protein backbone was superimposed closely to the *Mt*HCP_red_ structure (Supplementary Table S2). The hybrid cluster appeared to have both reduced and oxidised/semi-oxidised states. Therefore, the structure is referred to as *Mt*HCP_mix_ (Figure 4 and Supplementary Figure S4). A similar case of redox state mixture was reported for the HCP of *D. desulfuricans* (Macedo et al., 2003) (PDB code 1UPX). Both states of the hybrid cluster can be appropriately modelled due to the previous structural characterisation of HCPs. The reduced state is similar to the *Mt*HCP_red_ structure, with a noticeable difference in the glutamate 490 side chain position (Figures 4B,C). The carboxy group of the glutamate rotates 60° and disengages from the Fe atom (Fe number 8), leaving the Fe with three covalent bonds (Supplementary Figure S5). The oxidised/semi-oxidised state that contains a [4Fe μ_3_-S μ_2_-S, a persulphido and 3μ_2_-O] hybrid cluster fits very well with the ones observed in bacterial homologues. Here, the Fe atom 8 (according to the numbering from Hagen, 2022) is coordinated by two oxygen atoms, the glutamate 490 and the persulphido-cysteine 402 (Figures 4D,E). This “extracted” state will be referred to as *Mt*HCP_ox_ below.

**Figure 5:**
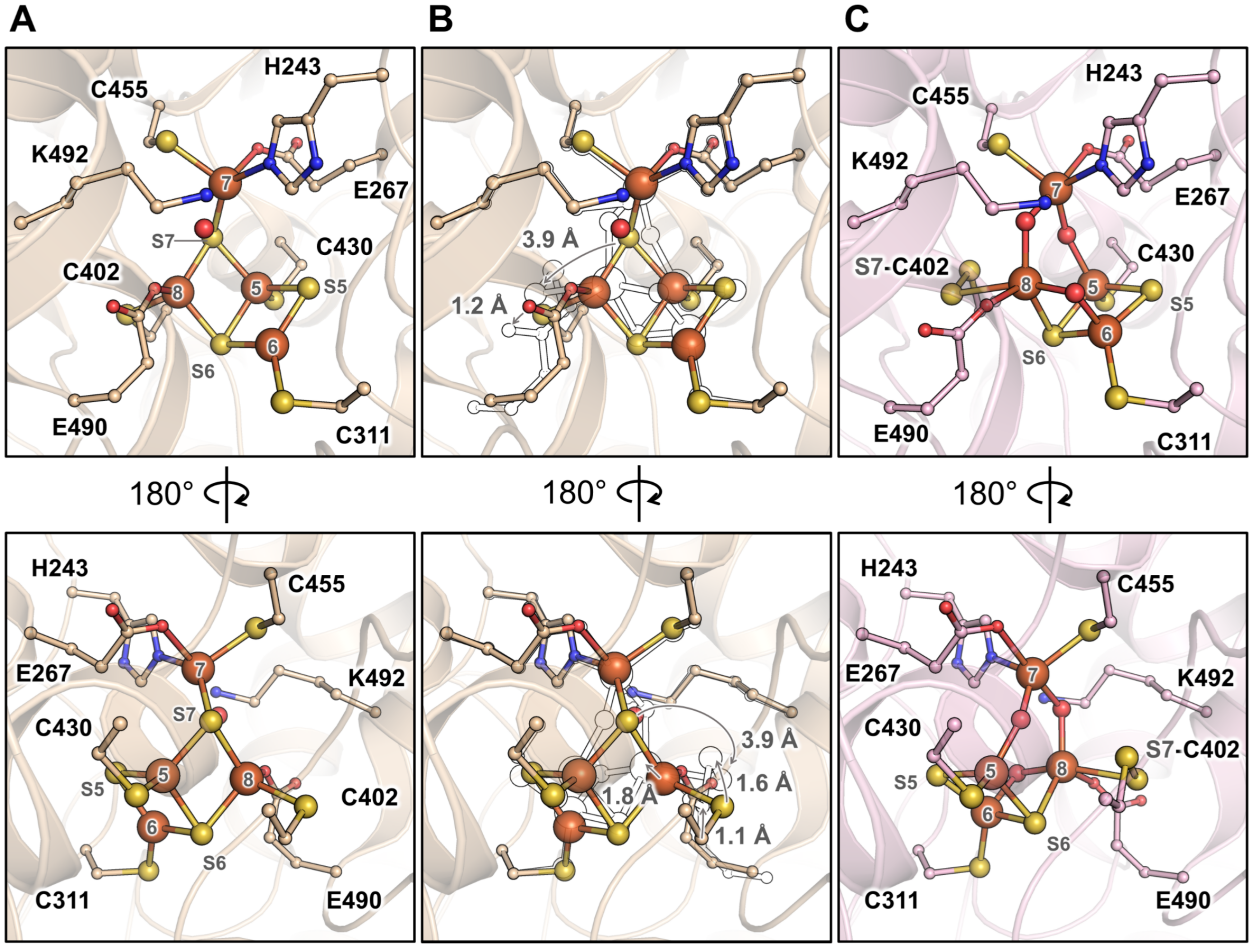
Transition of the hybrid cluster from reduced to oxidised/semi-oxidised state. **(A)** The reduced state structure *Mt*HCP_red_. **(B)** Transition from the reduced to oxidised/semi-oxidised states. The structure of the reduced state (as shown in panel **A**) is overlaid with the oxidised/semi-oxidised state outlined in black and white. The proposed movement of the atoms is symbolised by arrows, except for movements below 1 Å. **(C)** The oxidised/semi-oxidised state of the hybrid cluster (*Mt*HCP_ox_ extracted from *Mt*HCP_mix_). The structures are represented in wheat (reduced state) and light pink (oxidised/semi-oxidised) transparent cartoon, with the cofactors and residues coordinating/interacting with the cluster being shown as balls and sticks. Oxygen, nitrogen, sulphur, and iron atoms are coloured red, blue, yellow, and orange, respectively. The atom numbering originates from (Hagen, 2022).

The partial oxidation of the active site gives an opportunity to describe the conformational changes between reduced and oxidised states in *Mt*HCP (Figure 5). As for bacterial enzymes, the major changes are the position of Fe8 and S7 that undergo large movement (1.8 and 3.9 Å, respectively). Assuming a unique sulphur atom, the S7 position between Fe5 and Fe7 is replaced by oxygen and forms a persulphido group on the cysteine 402. The Fe8 loses the bonding with cysteine 402 and an oxygen bond the Fe6 and Fe8.

### 3.3. *Mt*HCP enzymatic activities

Because of their structural similarity, we aimed to compare the enzymatic properties of *Mt*HCP with that of previously characterised HCPs. The hydroxylamine reductase activity was assessed at 50°C, at a pH of 7.35, close to the physiological pH (Jarrell and Sprott, 1981), but also at pH 9.0 as tested in other studies, enabling a higher reaction turnover (Wolfe et al., 2002; Aragão et al., 2003; Cabello et al., 2004; Overeijnder et al., 2009). At pH 7.35, we determined an apparent *K*_M_ of 2.2 ± 0.5 mM, an apparent maximal velocity approximately 0.072 ± 0.004 μmol of hydroxylamine reduced/min/mg of purified HCP, and a turnover of 0.072 s^−1^ (Supplementary Figure S6). At pH 9.0, an apparent *K*_M_ of 3.9 ± 0.9 mM, *V*_max_ of 1.660 ± 0.157 μmol of hydroxylamine reduced/min/mg of purified HCP, and a turnover of 1.667 s^−1^ were obtained. Therefore, the enzyme exhibits an activity approximately 20–25-fold higher at pH 9.0, similar to the enzyme of *E. coli* (Wolfe et al., 2002) and in the range of other HCPs (Cabello et al., 2004; Overeijnder et al., 2009). However, purified *Mt*HCP exhibits a comparable *K*_M_, in the millimolar range, at both pHs, unlike the enzyme from *E. coli*. The kinetic parameters of the enzyme appear to be unsuitable with a physiological reduction of hydroxylamine, as for other HCPs (Hagen, 2022).

The peroxidase activity of *Mt*HCP was also tested but could not be monitored with the protocol used by Almeida and colleagues (Almeida et al., 2006). No significant enzyme-dependent ascorbate reduction could be recorded even at an enzyme concentration of 20 μM final. A significant enzyme-independent decrease in ascorbate absorbance occurred, especially at higher temperatures. As the specific peroxidase activities previously measured using *Ec*HCP and *Dd*HCP were relatively low [0.17 and 0.05 μmol.min^−1^.mg^−1^, respectively (Almeida et al., 2006)], a weak peroxidase activity of *Mt*HCP, possibly overlooked in our experiments, cannot be ruled out.

### 3.4. Hydrophobic tunnels

**Figure 6:**
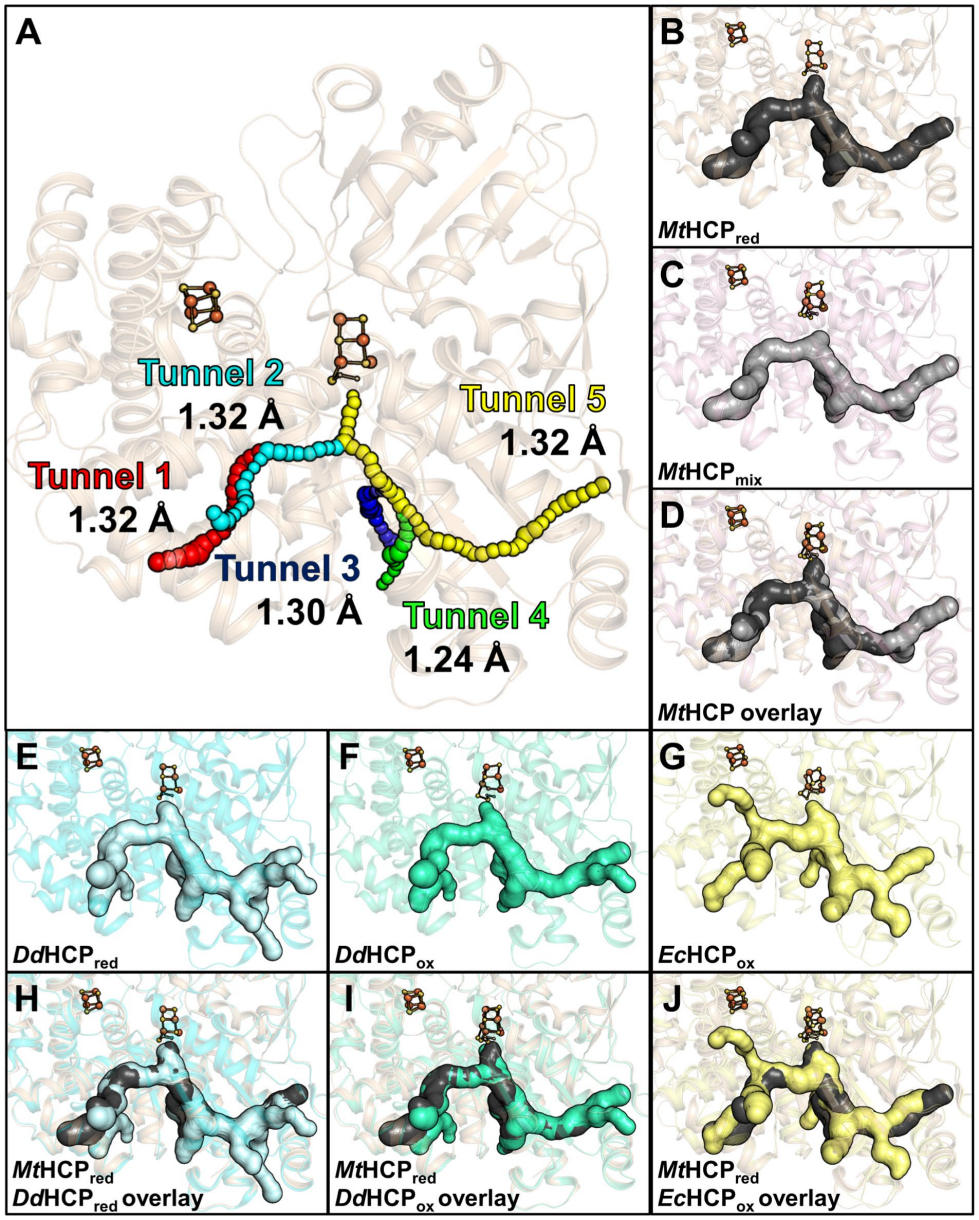
Channelling system in HCPs. **(A)** Tunnel analysis of *Mt*HCP_red_ by the *CAVER* programme. Spheres in different colours represent the tunnels predicted by the analysis, and the bottleneck radius is given in Ångström. **(B–D)** Prediction of the channelling system in *Mt*HCP structures. **(E–G)** Prediction of the channelling system in *Dd*HCP_red_ (PDB 1OA0), *Dd*HCP_ox_ (PDB 1GNL), and *Ec*HCP_ox_ (PDB 7DE4) structures, respectively. **(H–J)** Superimposition of *Mt*HCP_red_ and bacterial structures and their channelling systems. **(A–J)** The structures are represented as transparent cartoons. **(B–J)** The predicted channelling systems are in surfaces with the clusters, and the coordinating cysteine 402 (*Mt*HCP numbering) are presented in balls and sticks, with sulphur and iron coloured yellow and orange, respectively. The structures of *Mt*HCP_red_, *M*tHCP_mix_, *Dd*HCP_red_, *Dd*HCP_ox_, and *Ec*HCP_ox_ are coloured wheat, light pink, light cyan, green cyan, and light yellow, respectively.

The study of the first X-ray structure of an HCP already highlighted the presence of hydrophobic cavities within the enzyme structure (Cooper et al., 2000), Similarly to CODH (Lemaire and Wagner, 2021; Biester et al., 2022; Jeoung et al., 2022). A cavity analysis by the *CAVER* programme (Kozlikova et al., 2014) showed that the hydrophobic channels are also present in the *Mt*HCP_red_ structure (Figure 6A). Five channels devoid of any water molecules were detected, spanning an overall distance of approximately 63-Å (tunnel length from the extremities of tunnels 1 and 5) within the enzyme from an edge to another. The channelling system connects the active site to several openings on the protein surface, and most of the side chains structuring the channels are hydrophobic (57/62, see Supplementary Figures S2, S7), probably for repelling charged or polar molecules that would disturb the reaction. This is in agreement with the absence of water molecules in these tunnels. Depending on the tunnel, the bottleneck exhibits a radius ranging from 1.24 to 1.32 Å (Figure 5A). Analogous tunnels are predicted in the *Mt*HCP_mix_ structure, coherent with the structural conservation in both structures (Figures 6B–D and Supplementary Table S2). Noteworthy, tunnel 2 is obstructed by a molecule that we modelled as 2-methyl-pentane-2,4-diol based on the electron density (Supplementary Figure S8). As it is probably a non-physiological artefact of crystallisation, it was ignored for cavity prediction. Virtually identical tunnels are predicted in the structures of HCP homologues, with various additional extensions of tunnel 5 depending on the model used for the analysis (Figure 6 and Supplementary Figure S9). Most of these tunnels are conserved in the class II *Ec*HCP, but the slight difference in the position of the peptide chain (Supplementary Figure S3 and Supplementary Table S2) offsets the predicted channels (Figure 6).

As previously seen in CODH type V (Jeoung et al., 2022), the redox state of the hybrid cluster affects its access (Supplementary Figures S10A,B). Once in the oxidised state, the subtle changes in the hybrid cluster generate a so-called pinball effect to provoke the expulsion of the product, restraining the access to the cluster. *Mt*HCP obeys the same rule, narrowing the access to the hybrid cluster due to the formation of the persulphido-cysteine 402 (Supplementary Figures S10A,B). A similar movement occurs in *Dd*HCP and *Dv*HCP (Supplementary Figures S10C,D).

### 3.5. Phylogenetic position of *Mt*HCP

According to van Lis et al. (2020) study, most of the archaeal class I HCPs cluster in a single group containing bacterial sequences including DvHCP and DdHCP. This common branch suggests that this class of HCP was initially present in one of the domains and was acquired by the second via horizontal gene transfer (van Lis et al., 2020).

**Figure 7:**
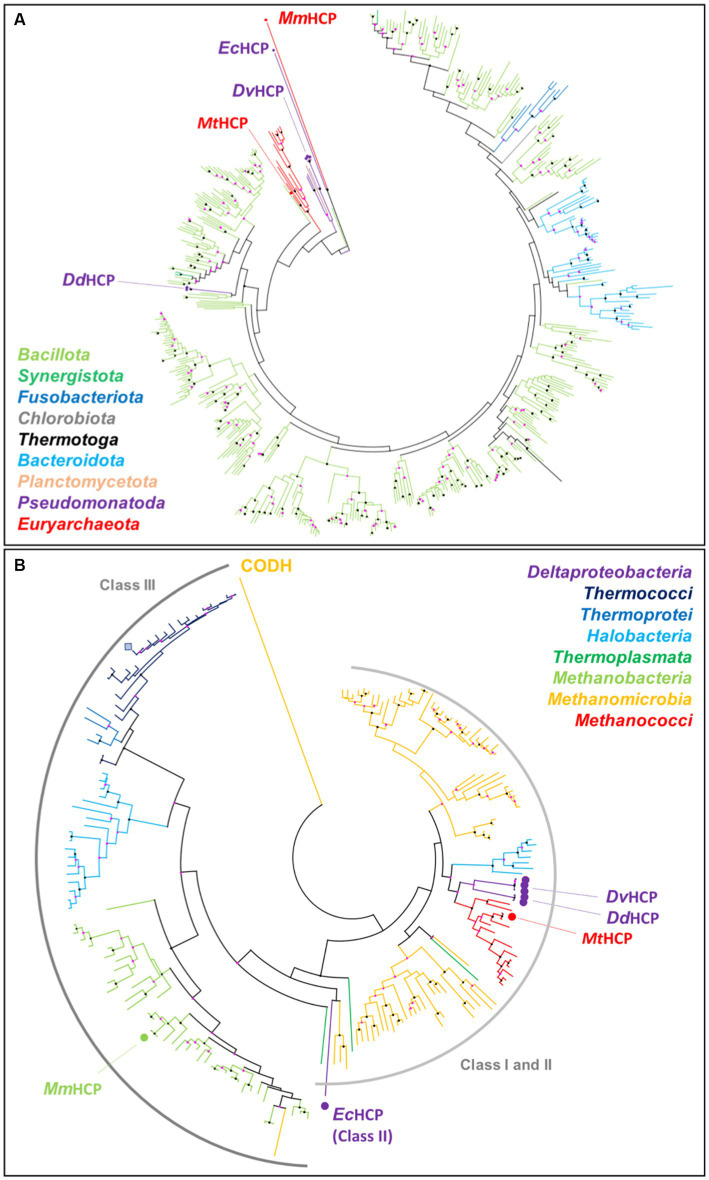
*Mt*HCP position in the phylogenetic tree of HCPs. **(A)** HCP phylogenetic tree from the PDB (highlighted by a dot and labelled) and the 500 closest sequences to *Mt*HCP from the RefSeq database. The branches are coloured by phylum. **(B)** HCP phylogenetic tree from the PDB (highlighted by a dot and labelled) and the 500 closest sequences to *Mt*HCP from the RefSeq database, restricted to the *Archaea* domain. The branches are coloured by class. The sequence of *P. furiosus* is marked as a blue square. The labelled CODH is used as an outgroup. Node statistics (200 replicates) between 50–90 and 90–100 are represented by pink and black dots, respectively.

A phylogenetic tree was constructed with the available sequences of HCPs from the Protein Data Bank and the 500 closest sequences to *Mt*HCP obtained by a BLAST analysis in the RefSeq database (Figure 7A). Over 500 obtained sequences, only 13 were from archaea, all from species belonging to the *Methanococcales* order. The remaining sequences were from bacterial class I enzymes belonging to different phyla, with a clear dominance of *Bacillota* (formerly *Firmicutes*). The archaeal sequences form a monophyletic group amongst the bacterial tree, and the sequences of the class II *Ec*HCP and the class III *Mm*HCP branch as outgroups of the generated class I HCPs tree. If the precise position of the archaeal HCPs amongst the bacterial sequences cannot be extracted from this analysis, it appears that *Mt*HCP and the enzymes from other *Methanococcales* belong to class I, as suggested by their sequences. In order to verify the suspicious position of the enzymes from *Methanococcales*, closer to bacterial HCPs than other archaeal species, another phylogenetic tree was constructed. The sequences used to construct the second tree were extracted from a similar BLAST analysis but restricted to the *Archaea* domain. The extracted sequences consisted of 184 sequences of archaeal HCPs, the remaining being annotated as CO-dehydrogenases. A preliminary tree showed that the CODH sequences were forming a completely separated branch and were discarded, a single sequence being kept as an outgroup (Figure 7B). This second tree gathers every archaeal HCP sequence from the RefSeq database. The class III HCPs, including *Mm*HCP and the enzyme from *P. furiosus*, form a separate branch, whilst the distinction between class I and II is unclear in this tree. This was expected because of the position of class II within the class I tree and because of the several ramifications of the class I phylogenetic tree (van Lis et al., 2020). The different branches mostly group organisms belonging to the same archaeal class, and some signs of horizontal gene transfer can be observed. For instance, a sequence of a class I HCP from a species belonging to the *Thermoplasmata* phylum groups in a branch gathering class I HCPs from *Methanomicrobia* species (Figure 7B). Similarly, the unique class III HCP found in the *Methanomicrobia* phylum groups in branch gathering enzymes from *Methanobacteria* (Figure 7B). The branch corresponding to *Methanococcales* is intriguingly close to that containing the class I HCPs from *Halobacteria*, yet *Methanococcales* and *Halobacteria* belong to a different clade (Adam et al., 2017). Moreover, the *Methanococcales* branch also forms a relatively clear group (node score of 77) with the sequences of bacterial class I HCP from *D. vulgaris* and *D. desulfuricans* (Figure 7B). It confirms the results of the previous BLAST analysis suggesting that *Mt*HCP and the other enzyme from *Methanococcales* are closer to bacterial enzymes than to any other archaeal HCP. This tendency is the same when both trees are gathered (Supplementary Figure S11).

## 4. Discussion

### 4.1. The HCP from *Methanococcales* as a sister group of characterised bacterial class I

The hybrid cluster protein is encoded in the genome of many anaerobic bacteria, archaea, and unicellular eukaryotes, including common laboratory models. Yet its function is still unclear. The singular chemistry of the cofactor has been intensively dissected in a few model *Pseudomonadota* species, but the enzyme has rarely been studied in archaea. The HCPs from *Methanococcales*, including the thermophilic methanogen *M. thermolithotrophicus*, belong to class I and branch closer to the HCPs extensively studied from *Desulfovibrio* species. It explains the singular sequence and structure conservation between *Dd*HCP, *Dv*HCP, and *Mt*HCP. Such a phylogenetic organisation led van Lis and colleagues to propose that this group of bacterial enzymes have an archaeal origin, acquired by the bacteria by inter-domain horizontal gene transfer (van Lis et al., 2020). The trees presented in Figure 7 and Supplementary Figure S11 are coherent with this proposal, as they show the branch of bacterial class I HCP emerging from the tree of archaeal HCPs. The HCPs from bacteria and *Methanococcales* species are harboured by the same branch in which they form monophyletic groups. This branch emerges from a tree mainly composed of sequences from *Methanomicrobia* and *Halobacteria*, yet belonging to a different archaeal clade compared to *Methanococcales*. Hence, the trees suggest horizontal gene transfer between different archaeal phyla but also from archaea to bacteria. As HCPs from *Methanococcales* species and bacteria cluster separately, one can hypothesise that these organisms acquired the gene from a similar archaeal ancestor or that the transfer of the protein from one group to the other occurred during their early evolution. Anyway, the proximity between archaeal and bacterial enzymes favours the hypothesis of a similar role within the organisms, coherently with the overall conservation of the active site architecture.

### 4.2. Physiological roles of HCP in methanogens

The different studies tend to agree on a physiological NO reductase role of HCP in bacteria, detoxifying the highly reactive gas in the provenance of side reactions of nitrate and nitrite reduction or from hosts in the case of pathogenic organisms. The NO reduction might be coupled with a regulatory protein trans-nitrosylation activity (Hagen, 2022). The similar kinetic parameters for the hydroxylamine reductase and the identical active site architecture support that *Mt*HCP, *Dd*HCP, and *Dv*HCP share catalytic properties. It also implies that *Mt*HCP should, in principle, perform NO reduction. However, most methanogens, especially *Methanococcales*, do not infect eukaryotic hosts, and only a few reduce nitrate (Belay et al., 1990; Jeanthon et al., 1998). In our culture conditions, *M. thermolithotrophicus* produced a sufficient quantity of HCP for native purification and crystallisation, yet the culture depends exclusively on ammonium as a nitrogen source. Accordingly, our recent transcriptomic analysis performed on *M. thermolithotrophicus* showed that the *hcp* gene expression is not impacted by the nitrogen source, unlike nearly all genes involved in nitrogen acquisition (Maslać et al., 2022). Therefore, it is rather less probable that *Mt*HCP is used for nitrate assimilation.

If *Mt*HCP physiologically serves as detoxifying NO reductase, one can thus wonder about the NO origin. Methanogens genomes do not harbour genes encoding for the NO-production pathway, such as NO synthase or NO-generating nitrite reductase. An alternative enzyme or pathway would be necessary. Nitrite reduction to ammonia can be performed by methanogens [e.g., by the F_420_-dependent sulfite/nitrite reductase (Jespersen et al., 2023)]. The enzymatic reduction of nitrite may, in theory, be a source of NO, similar to the nitrate reducers. Under these growing conditions, *M. thermolithotrophicus* does not have any exogenous nitrite source and therefore NO-production as a side product of nitrite reduction would depend on an endogenous nitrite source (e.g., by the anabolism), incoherent with the abundance of the HCP in the cytoplasm. An abiotic chemical reaction might occur in the sulfidic and metal-rich medium at 60°C as a source of nitrite or NO. However, numerous methanogens, including mesophilic species, contain a gene coding for HCP (class I or III). The protein was also shown to be relatively abundant in mesophilic *Bacillota* growing with ammonium as a nitrogen source (Valgepea et al., 2018). In the case of methanogens and other anaerobes, the natural abundance of the protein is, therefore, inadequate with the apparent absence of a notable biological or abiotic NO source. Either a source of NO escapes our understanding, or the HCP has an anabolic function more important and possibly more ancient than initially thought. Nitrate/nitrite-reducing organisms and pathogens would then have acquired or derived HCP to survive the NO concentrations encountered in situ. The HCPs from *Desulfovibrio* species accordingly branch separately in the bacterial HCP phylogenetic tree, which could indicate more recent inter-bacteria horizontal gene transfers (Supplementary Figure S11). Protein nitrosylation would be a plausible function for HCP in other anaerobes, yet it has not been shown to occur in archaea to our knowledge. Future studies will answer if protein nitrosylation has an important role in archaea and if the HCP is still involved in this process. Genetically tractable methanogens such as *Methanococcus maripaludis* (Susanti et al., 2019) or *Methanosarcina acetivorans* (Nayak and Metcalf, 2017) would be an appropriate tool for assessing this question.

### 4.3. Electron donors of the HCP

The electron donor for the reaction is unknown in class I HCPs. In contrast, the HCP reductase is the physiological partner of class II, providing the electrons for the reaction *via* NADH oxidation (Wang et al., 2016). Fujishiro and colleagues exemplified a possible role of the N-terminal insertion that would serve as a docking site to bind the HCP reductase in *Ec*HCP class II (Figure 2; Fujishiro et al., 2021). *Mt*HCP and its structural homologues do not present this extension or anything similar, and no gene coding for an HCP reductase exists in the organism. As is often the case for classes I and III HCPs, *Mt*HCP is encoded by an isolated gene with no related genes in the nearby genomic environment, which gives no indication of a putative partner. The systematic absence of other genes in synteny with *hcp* can be seen as an argument towards the involvement of a general electron donor. The reduced to semi oxidised state transition of the hybrid cluster exhibits a redox potential ranging from −250 to −150 mV in HCP class I, significantly lower than in class II (van Lis et al., 2020; Hagen, 2022). Assuming a similar redox potential in *Mt*HCP and approximately 40 mV lower potential for the [4Fe-4S] electron transferring cluster (van Lis et al., 2020), the enzyme reduction would be exergonic with NAD(P)H [*E*_0_´ = −320 mV (Walsh, 1986)] but also various substrates and electron shuttles of *M. thermolithotrophicus* such as ferredoxin [physiological potential approximately −500 mV (Schuchmann and Müller, 2014)] or F_420_ [*E*_0_´ = −360 mV (Walsh, 1986)]. If the HCP reduction by ferredoxin would be largely exergonic, it would also represent a substantial energy loss, improbable in such an energy-limited organism, whilst coupling the reaction with NAD(P)H and F_420_H_2_ would require an oxidase partner.

The role of the hydrated polar cavity connecting the cubane [4Fe-4S] cluster to the surface is unknown. Using soluble molecules that would deliver electrons directly to the cluster would require a catalytic mechanism that has not been described yet. Since such cubane clusters are considered to serve as electron relays rather than catalytic centres, this hypothesis is unlikely. Furthermore, a similar cavity is present in *Ec*HCP that is reduced by another protein (Figure 2). It could also affect the structural stabilisation or regulation of enzyme activity.

## 5. Conclusion

The last years of research highlighted the NO reductase activity of HCP, used for NO detoxification and protein nitrosylation. If this is in perfect agreement with the enzyme production during nitrate and nitrite reduction or a defence mechanism during pathogenicity, its role is elusive in bacteria and archaea growing without nitrate, nitrite, or any specific source of NO. That is the case of the methanogen *M. thermolithotrophicus*, abundantly producing its HCP. The structural characterisation of this enzyme highlighted a striking similarity with bacterial enzymes, and for a good reason: HCPs from *Methanococcales* are closest to bacterial HCPs than to any others. As the growth conditions contain no apparent source of NO, it is plausible that the HCP is not used for NO detoxification but rather for an important and overlooked function in the anabolism of methanogens and probably in acetogens or other *Bacillota* species. Future genetic studies on model organisms will have to pursue this quest to extend the physiological role of HCPs in anaerobes.

## Data availability statement

The data presented in the study are deposited in the PDB repository, accession number 8CNR and 8CNS.

## Author contributions

OL and TW designed the research, collected the X-ray data, solved the structures, built the models, and analysed the structures. OL purified and crystallised the proteins. MB performed the activity measurements, electrophoreses, and size-exclusion chromatography experiments. OL and MB performed the bioinformatics analyses. All authors participated in the manuscript writing.

## Funding

This study was funded by the Max-Planck-Gesellschaft and the Deutsche Forschungsgemeinschaft priority programme 1927, “Iron-Sulfur for Life” WA 4053/1-1.

## Conflict of interest

The authors declare that the research was conducted in the absence of any commercial or financial relationships that could be construed as a potential conflict of interest.

## Publisher’s note

All claims expressed in this article are solely those of the authors and do not necessarily represent those of their affiliated organizations, or those of the publisher, the editors and the reviewers. Any product that may be evaluated in this article, or claim that may be made by its manufacturer, is not guaranteed or endorsed by the publisher.

## Footnotes

^1^ http://molprobity.biochem.duke.edu

## References

[ref1] Adam P. S. Borrel G. Brochier-Armanet C. Gribaldo S. (2017). The growing tree of Archaea: new perspectives on their diversity, evolution and ecology. ISME J. 11, 2407–2425. doi: 10.1038/ismej.2017.122, PMID: 28777382 PMC5649171

[ref2] Almeida C. C. Romão C. V. Lindley P. F. Teixeira M. Saraiva L. M. (2006). The role of the hybrid cluster protein in oxidative stress defense. J. Biol. Chem. 281, 32445–32450. doi: 10.1074/jbc.M605888200, PMID: 16928682

[ref3] Altschul S. F. Madden T. L. Schäffer A. A. Zhang J. Zhang Z. Miller W.. (1997). Gapped BLAST and PSI-BLAST: a new generation of protein database search programs. Nucleic Acids Res. 25, 3389–3402. doi: 10.1093/nar/25.17.3389, PMID: 9254694 PMC146917

[ref4] Aragão D. Macedo S. Mitchell E. P. Romão C. V. Liu M. Y. Frazão C.. (2003). Reduced hybrid cluster proteins (HCP) from *Desulfovibrio desulfuricans* ATCC 27774 and *Desulfovibrio vulgaris* (Hildenborough): X-ray structures at high resolution using synchrotron radiation. J. Biol. Inorganic Chem. 8, 540–548. doi: 10.1007/s00775-003-0443-x, PMID: 12764602

[ref5] Aragão D. Mitchell E. P. Frazão C. F. Carrondo M. A. Lindley P. F. (2008). Structural and functional relationships in the hybrid cluster protein family: structure of the anaerobically purified hybrid cluster protein from *Desulfovibrio vulgaris* at 1.35 Å resolution. Acta Crystallogr. Sect. D 64, 665–674. doi: 10.1107/S0907444908009165, PMID: 18560155

[ref6] Belay N. Jung K.-Y. Rajagopal B. S. Kremer J. D. Daniels L. (1990). Nitrate as a sole nitrogen source for *Methanococcus thermolithotrophicus* and its effect on growth of several methanogenic bacteria. Curr. Microbiol. 21, 193–198. doi: 10.1007/BF02092121

[ref7] Beliaev A. S. Klingeman D. M. Klappenbach J. A. Wu L. Romine M. F. Tiedje J. M.. (2005). Global transcriptome analysis of *Shewanella oneidensis* MR-1 exposed to different terminal electron acceptors. J. Bacteriol. 187, 7138–7145. doi: 10.1128/JB.187.20.7138-7145.2005, PMID: 16199584 PMC1251602

[ref8] Biester A. Dementin S. Drennan C. L. (2022). Visualizing the gas channel of a monofunctional carbon monoxide dehydrogenase. J. Inorg. Biochem. 230:111774. doi: 10.1016/j.jinorgbio.2022.111774, PMID: 35278753 PMC9093221

[ref9] Briolat V. Reysset G. (2002). Identification of the *Clostridium perfringens* genes involved in the adaptive response to oxidative stress. J. Bacteriol. 184, 2333–2343. doi: 10.1128/JB.184.9.2333-2343.2002, PMID: 11948145 PMC134984

[ref10] Bulot S. Audebert S. Pieulle L. Seduk F. Baudelet E. Espinosa L.. (2019). Clustering as a means to control nitrate respiration efficiency and toxicity in *Escherichia coli*. mBio 10:e01832-19. doi: 10.1128/mBio.01832-19, PMID: 31641084 PMC6805990

[ref11] Cabello P. Pino C. Olmo-Mira M. F. Castillo F. Roldán M. D. Moreno-Vivián C. (2004). Hydroxylamine assimilation by *Rhodobacter capsulatus* E1F1. Requirement of the *hcp* gene (hybrid cluster protein) located in the nitrate assimilation *nas* gene region for hydroxylamine reduction. J. Biol. Chem. 279, 45485–45494. doi: 10.1074/jbc.M404417200, PMID: 15322098

[ref12] Cadby I. T. Faulkner M. Cheneby J. Long J. van Helden J. Dolla A.. (2017). Coordinated response of the *Desulfovibrio desulfuricans* 27774 transcriptome to nitrate, nitrite and nitric oxide. Sci. Rep. 7:16228. doi: 10.1038/s41598-017-16403-4, PMID: 29176637 PMC5701242

[ref13] Chen V. B. Arendall W. B. 3rd Headd J. J. Keedy D. A. Immormino R. M. Kapral G. J.. (2010). Mol Probity: all-atom structure validation for macromolecular crystallography. Acta Crystallogr. D Biol. Crystallogr. 66, 12–21. doi: 10.1107/S0907444909042073, PMID: 20057044 PMC2803126

[ref14] Cole J. A. (2021). Anaerobic bacterial response to nitric oxide stress: widespread misconceptions and physiologically relevant responses. Mol. Microbiol. 116, 29–40. doi: 10.1111/mmi.14713, PMID: 33706420

[ref15] Cooper S. J. Garner C. D. Hagen W. R. Lindley P. F. Bailey S. (2000). Hybrid-cluster protein (HCP) from *Desulfovibrio vulgaris* (Hildenborough) at 1.6 Å resolution. Biochemistry 39, 15044–15054. doi: 10.1021/bi001483m 11106482

[ref16] Edgar R. C. (2004). MUSCLE: a multiple sequence alignment method with reduced time and space complexity. BMC Bioinformat. 5:113. doi: 10.1186/1471-2105-5-113, PMID: 15318951 PMC517706

[ref17] Emsley P. Lohkamp B. Scott W. G. Cowtan K. (2010). Features and development of *Coot*. Acta Crystallogr. D Biol. Crystallogr. 66, 486–501. doi: 10.1107/S0907444910007493, PMID: 20383002 PMC2852313

[ref18] Faeder E. J. Davis P. S. Siegel L. M. (1974). Reduced nicotinamide adenine dinucleotide phosphate-sulfite reductase of enterobacteria: V. STUDIES WITH THE ESCHERICHIA COLI HEMOFLAVOPROTEIN DEPLETED OF FLAVIN MONONUCLEOTIDE: DISTINCT ROLES FOR THE FLAVIN ADENINE DINUCLEOTIDE AND FLAVIN MONONUCLEOTIDE PROSTHETIC GROUPS IN CATALYSIS. J. Biol. Chem. 249, 1599–1609. doi: 10.1016/S0021-9258(19)42923-2, PMID: 4150392

[ref19] Filenko N. A. Browning D. F. Cole J. A. (2005). Transcriptional regulation of a hybrid cluster (prismane) protein. Biochem. Soc. Trans. 33, 195–197. doi: 10.1042/BST0330195 15667305

[ref20] Filenko N. Spiro S. Browning D. F. Squire D. Overton T. W. Cole J.. (2007). The NsrR regulon of *Escherichia coli* K-12 includes genes encoding the hybrid cluster protein and the periplasmic, respiratory nitrite reductase. J. Bacteriol. 189, 4410–4417. doi: 10.1128/JB.00080-07, PMID: 17449618 PMC1913375

[ref21] Fujishiro T. Ooi M. Takaoka K. (2021). Crystal structure of *Escherichia coli* class II hybrid cluster protein, HCP, reveals a [4Fe-4S] cluster at the N-terminal protrusion. FEBS J. 288, 6752–6768. doi: 10.1111/febs.16062 34101368

[ref22] Fujishiro T. Takaoka K. (2022). Class III hybrid cluster protein (HCP) from *Methanothermobacter marburgensis*. Jpn. Soc. Prom. Sci. doi: 10.2210/pdb7E0L/pdb

[ref23] Gao S.-H. Ho J. Y. Fan L. Nouwens A. Hoelzle R. D. Schulz B.. (2019). A comparative proteomic analysis of *Desulfovibrio vulgaris* Hildenborough in response to the antimicrobial agent free nitrous acid. Sci. Total Environ. 672, 625–633. doi: 10.1016/j.scitotenv.2019.03.442, PMID: 30974354

[ref24] Hagen W. R. (2019). EPR spectroscopy of putative enzyme intermediates in the NO reductase and the auto-nitrosylation reaction of *Desulfovibrio vulgaris* hybrid cluster protein. FEBS Lett. 593, 3075–3083. doi: 10.1002/1873-3468.13539 31318443

[ref25] Hagen W. R. (2022). Structure and function of the hybrid cluster protein. Coord. Chem. Rev. 457:214405. doi: 10.1016/j.ccr.2021.214405

[ref26] Hagen W. R. Pierik A. J. Veeger C. (1989). Novel electron paramagnetic resonance signals from an Fe/S protein containing six iron atoms. J. Chem. Soc. Faraday Trans. 1 85, 4083–4090.

[ref27] Hagen W. R. van den Berg W. A. van Dongen W. M. Reijerse E. J. van Kan P. J. (1998). EPR spectroscopy of biological iron–sulfur clusters with spin-admixed *S*= 3/2. J. Chem. Soc. Faraday Trans. 94, 2969–2973. doi: 10.1039/a803059f

[ref28] Haveman S. A. Greene E. A. Stilwell C. P. Voordouw J. K. Voordouw G. (2004). Physiological and gene expression analysis of inhibition of *Desulfovibrio vulgaris* Hildenborough by nitrite. J. Bacteriol. 186, 7944–7950. doi: 10.1128/JB.186.23.7944-7950.2004, PMID: 15547266 PMC529081

[ref29] He Q. Huang K. H. He Z. Alm E. J. Fields M. W. Hazen T. C.. (2006). Energetic consequences of nitrite stress in *Desulfovibrio vulgaris* Hildenborough, inferred from global transcriptional analysis. Appl. Environ. Microbiol. 72, 4370–4381. doi: 10.1128/AEM.02609-05, PMID: 16751553 PMC1489655

[ref30] Heo J. Wolfe M. T. Staples C. R. Ludden P. W. (2002). Converting the NiFeS carbon monoxide dehydrogenase to a hydrogenase and a hydroxylamine reductase. J. Bacteriol. 184, 5894–5897. doi: 10.1128/JB.184.21.5894-5897.2002, PMID: 12374822 PMC135374

[ref31] Jarrell K. F. Sprott G. D. (1981). The transmembrane electrical potential and intracellular pH in methanogenic bacteria. Can. J. Microbiol. 27, 720–728. doi: 10.1139/m81-110, PMID: 7296406

[ref32] Jeanthon C. L’Haridon S. Reysenbach A. L. Vernet M. Messner P. Sleytr U. B.. (1998). *Methanococcus infernus* sp. nov., a novel hyperthermophilic lithotrophic methanogen isolated from a deep-sea hydrothermal vent. Int. J. Syst. Evol. Microbiol. 48, 913–919. 10.1099/00207713-48-3-913 9734046

[ref33] Jeoung J.-H. Fesseler J. Domnik L. Klemke F. Sinnreich M. Teutloff C.. (2022). A morphing [4Fe-3S-nO]-cluster within a carbon monoxide dehydrogenase scaffold. Angew. Chem. Int. Ed. 61:e202117000. doi: 10.1002/anie.202117000, PMID: 35133707 PMC9311411

[ref34] Jespersen M. Pierik A. J. Wagner T. (2023). Structures of the sulfite detoxifying F_420_-dependent enzyme from *Methanococcales*. Nat. Chem. Biol. doi: 10.1038/s41589-022-01232-y, PMID: 36658338 PMC10229431

[ref35] Kim C. C. Monack D. Falkow S. (2003). Modulation of virulence by two acidified nitrite-responsive loci of *Salmonella enterica* Serovar Typhimurium. Infect. Immun. 71, 3196–3205. doi: 10.1128/IAI.71.6.3196-3205.2003, PMID: 12761099 PMC155741

[ref36] Klüber H. D. Conrad R. (1998). Inhibitory effects of nitrate, nitrite, NO and N_2_O on methanogenesis by *Methanosarcina barkeri* and *Methanobacterium bryantii*. FEMS Microbiol. Ecol. 25, 331–339. doi: 10.1016/S0168-6496(97)00102-5

[ref37] Kozlikova B. Sebestova E. Sustr V. Brezovsky J. Strnad O. Daniel L.. (2014). CAVER analyst 1.0: graphic tool for interactive visualization and analysis of tunnels and channels in protein structures. Bioinformatics 30, 2684–2685. doi: 10.1093/bioinformatics/btu364, PMID: 24876375

[ref38] Krissinel E. Henrick K. (2004). Secondary-structure matching (SSM), a new tool for fast protein structure alignment in three dimensions. Acta Crystallogr. D Biol. Crystallogr. 60, 2256–2268. doi: 10.1107/S0907444904026460, PMID: 15572779

[ref39] Kröckel M. Trautwein A. X. Arendsen A. F. Hagen W. R. (1998). The prismane protein resolved--Mössbauer investigation of a 4Fe cluster with an unusual mixture of bridging ligands and metal coordinations. Eur. J. Biochem. 251, 454–461. doi: 10.1046/j.1432-1327.1998.2510454.x, PMID: 9492318

[ref40] Kumar S. Stecher G. Li M. Knyaz C. Tamura K. (2018). MEGA X: molecular evolutionary genetics analysis across computing platforms. Mol. Biol. Evol. 35, 1547–1549. doi: 10.1093/molbev/msy096, PMID: 29722887 PMC5967553

[ref41] Lemaire O. N. Infossi P. Ali Chaouche A. Espinosa L. Leimkühler S. Giudici-Orticoni M. T.. (2018). Small membranous proteins of the TorE/NapE family, crutches for cognate respiratory systems in *Proteobacteria*. Sci. Rep. 8:13576. doi: 10.1038/s41598-018-31851-2, PMID: 30206249 PMC6134056

[ref42] Lemaire O. N. Wagner T. (2021). Gas channel rerouting in a primordial enzyme: structural insights of the carbon-monoxide dehydrogenase/acetyl-CoA synthase complex from the acetogen *Clostridium autoethanogenum*. Biochim. Biophys. Acta Bioenerg. 1862:148330. doi: 10.1016/j.bbabio.2020.148330 33080205

[ref43] Liebschner D. Afonine P. V. Baker M. L. Bunkóczi G. Chen V. B. Croll T. I.. (2019). Macromolecular structure determination using X-rays, neutrons and electrons: recent developments in *Phenix*. Acta Crystallogr. D Struct. Biol. 75, 861–877. doi: 10.1107/S2059798319011471, PMID: 31588918 PMC6778852

[ref44] Macedo S. Aragão D. Mitchell E. P. Lindley P. (2003). Structure of the hybrid cluster protein (HCP) from *Desulfovibrio desulfuricans* ATCC 27774 containing molecules in the oxidized and reduced states. Acta Crystallogr. D Biol. Crystallogr. 59, 2065–2071. doi: 10.1107/S0907444903025861, PMID: 14646063

[ref45] Macedo S. Mitchell E. P. Romão C. V. Cooper S. J. Coelho R. Liu M. Y.. (2002). Hybrid cluster proteins (HCPs) from *Desulfovibrio desulfuricans* ATCC 27774 and *Desulfovibrio vulgaris* (Hildenborough): X-ray structures at 1.25 Å resolution using synchrotron radiation. J. Biol. Inorg. Chem. 7, 514–525. doi: 10.1007/s00775-001-0326-y, PMID: 11941509

[ref46] Mager J. (1960). A TPNH-linked sulfite reductase and its relation to hydroxylamine reductase in *Enterobacteriaceae*. Biochim. Biophys. Acta 41, 553–555. doi: 10.1016/0006-3002(60)90065-2 14419795

[ref47] Marritt S. J. Farrar J. A. Breton J. L. Hagen W. R. Thomson A. J. (1995). Characterization of the prismane protein from *Desulfovibrio vulgaris* (Hildenborough) by low-temperature magnetic circular dichroic spectroscopy. Eur. J. Biochem. 232, 501–505. doi: 10.1111/j.1432-1033.1995.501zz.x, PMID: 7556199

[ref48] Maslać N. Sidhu C. Teeling H. Wagner T. (2022). Comparative transcriptomics sheds light on remodeling of gene expression during diazotrophy in the thermophilic methanogen *Methanothermococcus thermolithotrophicus*. mBio 13:e0244322. doi: 10.1128/mbio.02443-22, PMID: 36409126 PMC9765008

[ref49] Moura I. Tavares P. Moura J. J. Ravi N. Huynh B. H. Liu M. Y.. (1992). Direct spectroscopic evidence for the presence of a 6Fe cluster in an iron-sulfur protein isolated from *Desulfovibrio desulfuricans* (ATCC 27774). J. Biol. Chem. 267, 4489–4496. doi: 10.1016/S0021-9258(18)42859-1, PMID: 1311311

[ref50] Nayak D. D. Metcalf W. W. (2017). Cas9-mediated genome editing in the methanogenic archaeon *Methanosarcina acetivorans*. Proc. Natl. Acad. Sci. U. S. A. 114, 2976–2981. doi: 10.1073/pnas.1618596114, PMID: 28265068 PMC5358397

[ref51] Okinaka Y. Yang C. H. Perna N. T. Keen N. T. (2002). Microarray profiling of *Erwinia chrysanthemi* 3937 genes that are regulated during plant infection. Mol. Plant Microbe Interact. 15, 619–629. doi: 10.1094/MPMI.2002.15.7.619, PMID: 12118877

[ref52] Ostrowski J. Barber M. J. Rueger D. C. Miller B. E. Siegel L. M. Kredich N. M. (1989). Characterization of the flavoprotein moieties of NADPH-sulfite reductase from *Salmonella typhimurium* and *Escherichia coli*. Physicochemical and catalytic properties, amino acid sequence deduced from DNA sequence of *cysJ*, and comparison with NADPH-cytochrome P-450 reductase. J. Biol. Chem. 264, 15796–15808. doi: 10.1016/S0021-9258(18)71547-0, PMID: 2550423

[ref53] Overeijnder M. L. Hagen W. R. Hagedoorn P.-L. (2009). A thermostable hybrid cluster protein from *Pyrococcus furiosus*: effects of the loss of a three helix bundle subdomain. J. Biol. Inorganic Chem. 14, 703–710. doi: 10.1007/s00775-009-0483-y, PMID: 19241093 PMC2694928

[ref54] Pereira A. S. Tavares P. Krebs C. Huynh B. H. Rusnak F. Moura I.. (1999). Biochemical and spectroscopic characterization of overexpressed fuscoredoxin from *Escherichia coli*. Biochem. Biophys. Res. Commun. 260, 209–215. doi: 10.1006/bbrc.1999.0748, PMID: 10381368

[ref55] Pierik A. J. Hagen W. R. Dunham W. R. Sands R. H. (1992). Multi-frequency EPR and high-resolution Mössbauer spectroscopy of a putative [6Fe-6S] prismane-cluster-containing protein from *Desulfovibrio vulgaris* (Hildenborough). Characterization of a supercluster and superspin model protein. Eur. J. Biochem. 206, 705–719. doi: 10.1111/j.1432-1033.1992.tb16977.x, PMID: 1318833

[ref56] Pierik A. J. Wolbert R. B. Mutsaers P. H. Hagen W. R. Veeger C. (1992). Purification and biochemical characterization of a putative [6Fe-6S] prismane-cluster-containing protein from *Desulfovibrio vulgaris* (Hildenborough). Eur. J. Biochem. 206, 697–704. doi: 10.1111/j.1432-1033.1992.tb16976.x, PMID: 1318832

[ref57] Robert X. Gouet P. (2014). Deciphering key features in protein structures with the new ENDscript server. Nucleic Acids Res. 42, W320–W324. doi: 10.1093/nar/gku316 24753421 PMC4086106

[ref58] Roos V. Klemm P. (2006). Global gene expression profiling of the asymptomatic bacteriuria *Escherichia coli* strain 83972 in the human urinary tract. Infect. Immun. 74, 3565–3575. doi: 10.1128/IAI.01959-05, PMID: 16714589 PMC1479258

[ref59] Schuchmann K. Müller V. (2014). Autotrophy at the thermodynamic limit of life: a model for energy conservation in acetogenic bacteria. Nat. Rev. Microbiol. 12, 809–821. doi: 10.1038/nrmicro3365 25383604

[ref60] Seth D. Hess D. T. Hausladen A. Wang L. Wang Y.-J. Stamler J. S. (2018). A multiplex enzymatic machinery for cellular protein S-nitrosylation. Mol. Cell 69, 451–464.e6. doi: 10.1016/j.molcel.2017.12.025, PMID: 29358078 PMC5999318

[ref61] Sievers F. Wilm A. Dineen D. Gibson T. J. Karplus K. Li W.. (2011). Fast, scalable generation of high-quality protein multiple sequence alignments using Clustal Omega. Mol. Syst. Biol. 7:539. doi: 10.1038/msb.2011.75, PMID: 21988835 PMC3261699

[ref62] Spiro S. (2007). Regulators of bacterial responses to nitric oxide. FEMS Microbiol. Rev. 31, 193–211. doi: 10.1111/j.1574-6976.2006.00061.x 17313521

[ref63] Stokkermans J. P. W. G. Houba P. H. J. Pierik A. J. Hagen W. R. van Dongen W. M. A. M. Veeger C. (1992). Overproduction of prismane protein in *Desulfovibrio vulgaris* (Hildenborough): evidence for a second *S*= 1/2-spin system in the one-electron reduced state. Eur. J. Biochem. 210, 983–988. doi: 10.1111/j.1432-1033.1992.tb17503.x, PMID: 1336462

[ref64] Susanti D. Frazier M. C. Mukhopadhyay B. (2019). A genetic system for *Methanocaldococcus jannaschii*: an evolutionary deeply rooted hyperthermophilic methanarchaeon. Front. Microbiol.:1256:10. doi: 10.3389/fmicb.2019.01256 31333590 PMC6616113

[ref65] Tavares P. Pereira A. S. Krebs C. Ravi N. Moura J. J. Moura I.. (1998). Spectroscopic characterization of a novel tetranuclear Fe cluster in an iron-sulfur protein isolated from *Desulfovibrio desulfuricans*. Biochemistry 37, 2830–2842. doi: 10.1021/bi9723008, PMID: 9485434

[ref66] Tickle IJ Flensburg C Keller P Paciorek W Sharff A Vonrhein C. STARANISO (Cambridge, United Kingdom: Global Phasing), (2018).

[ref67] Usón I. Sheldrick G. (2018). An introduction to experimental phasing of macromolecules illustrated by *SHELX*; new autotracing features. Acta Crystallogr. Sec. D Struct. Biol. 74, 106–116. doi: 10.1107/S2059798317015121, PMID: 29533236 PMC5947774

[ref68] Valgepea K. De Souza Pinto Lemgruber R. Abdalla T. Binos S. Takemori N. Takemori A.. (2018). H_2_ drives metabolic rearrangements in gas-fermenting *Clostridium autoethanogenum*. Biotechnol. Biofuels 11:55. doi: 10.1186/s13068-018-1052-9 29507607 PMC5831606

[ref69] van den Berg W. A. Hagen W. R. van Dongen W. M. (2000). The hybrid-cluster protein (‘prismane protein’) from *Escherichia coli*. Characterization of the hybrid-cluster protein, redox properties of the [2Fe-2S] and [4Fe-2S-2O] clusters and identification of an associated NADH oxidoreductase containing FAD and [2Fe-2S]. Eur. J. Biochem. 267, 666–676. doi: 10.1046/j.1432-1327.2000.01032.x, PMID: 10651802

[ref70] van den Berg W. A. Stevens A. A. Verhagen M. F. van Dongen W. M. Hagen W. R. (1994). Overproduction of the prismane protein from *Desulfovibrio desulfuricans* ATCC 27774 in *Desulfovibrio vulgaris* (Hildenborough) and EPR spectroscopy of the [6Fe-6S] cluster in different redox states. Biochim. Biophys. Acta Protein Struct. Mol. Enzymol. 1206, 240–246. doi: 10.1016/0167-4838(94)90214-3, PMID: 8003528

[ref71] van Lis R. Brugière S. Baffert C. Couté Y. Nitschke W. Atteia A. (2020). Hybrid cluster proteins in a photosynthetic microalga. FEBS J. 287, 721–735. doi: 10.1111/febs.15025, PMID: 31361397

[ref72] Vonrhein C. Flensburg C. Keller P. Sharff A. Smart O. Paciorek W.. (2011). Data processing and analysis with the *autoPROC* toolbox. Acta Crystallogr. Sect. D 67, 293–302. doi: 10.1107/S0907444911007773, PMID: 21460447 PMC3069744

[ref73] Walsh C. (1986). Naturally occurring 5-deazaflavin coenzymes: biological redox roles. Acc. Chem. Res. 19, 216–221. doi: 10.1021/ar00127a004

[ref74] Wang Y. Cai X. Fan J. Wang D. Mao Y. (2022). Transcriptome analysis provides new insights into the tolerance and aerobic reduction of *Shewanella decolorationis* Ni1-3 to bromate. Appl. Microbiol. Biotechnol. 106, 4749–4761. doi: 10.1007/s00253-022-12006-w, PMID: 35708750

[ref75] Wang J. Vine C. E. Balasiny B. K. Rizk J. Bradley C. L. Tinajero-Trejo M.. (2016). The roles of the hybrid cluster protein, Hcp and its reductase, Hcr, in high affinity nitric oxide reduction that protects anaerobic cultures of *Escherichia coli* against nitrosative stress. Mol. Microbiol. 100, 877–892. doi: 10.1111/mmi.13356, PMID: 26879449

[ref76] Winn M. D. Ballard C. C. Cowtan K. D. Dodson E. J. Emsley P. Evans P. R.. (2011). Overview of the *CCP*4 suite and current developments. Acta Crystallogr. D Biol. Crystallogr. 67, 235–242. doi: 10.1107/S0907444910045749, PMID: 21460441 PMC3069738

[ref77] Wolfe M. T. Heo J. Garavelli J. S. Ludden P. W. (2002). Hydroxylamine reductase activity of the hybrid cluster protein from *Escherichia coli*. J. Bacteriol. 184, 5898–5902. doi: 10.1128/JB.184.21.5898-5902.2002, PMID: 12374823 PMC135376

[ref78] Yu Z. Pesesky M. Zhang L. Huang J. Winkler M. Chistoserdova L. (2020). A complex interplay between nitric oxide, quorum sensing, and the unique secondary metabolite tundrenone constitutes the hypoxia response in *Methylobacter*. mSystems 5:e00770-19. doi: 10.1128/mSystems.00770-19 31964770 PMC6977074

[ref79] Yurkiw M. A. Voordouw J. Voordouw G. (2012). Contribution of rubredoxin: oxygen oxidoreductases and hybrid cluster proteins of *Desulfovibrio vulgaris* Hildenborough to survival under oxygen and nitrite stress. Environ. Microbiol. 14, 2711–2725. doi: 10.1111/j.1462-2920.2012.02859.x, PMID: 22947039

[ref80] Zhang W. Culley D. E. Hogan M. Vitiritti L. Brockman F. J. (2006). Oxidative stress and heat-shock responses in *Desulfovibrio vulgaris* by genome-wide transcriptomic analysis. Antonie Van Leeuwenhoek 90, 41–55. doi: 10.1007/s10482-006-9059-9, PMID: 16680520

