# Supplementary material for: Structural and biochemical elucidation of class I hybrid cluster protein natively extracted from a marine methanogenic archaeon
Source: Front Microbiol. 2023 May 11;14:1179204. doi: 10.3389/fmicb.2023.1179204 (PMC10210160; doi:10.3389/fmicb.2023.1179204)
Supplement: Supplementary file 1 [file Data_Sheet_1.docx]

Structural and biochemical elucidation of class I hybrid cluster protein natively extracted from a marine methanogenic archaeon

Olivier N Lemaire^*^, Mélissa Belhamri, Tristan Wagner^*^

Max Planck Institute for Marine Microbiology, Celsiusstraße 1, 28359 Bremen, Germany.

*** Correspondence:**Dr. Olivier N Lemaire

[olemaire@mpi-bremen.de](mailto:olemaire@mpi-bremen.de)

Dr. Tristan Wagner
[twagner@mpi-bremen.de](mailto:twagner@mpi-bremen.de)

Supplementary Material

**Table S1.** X-ray analysis statistics

**Table S2.** Root mean square deviation (r.m.s.d.) and sequence identity between the *Mt*HCP structures and the structurally characterised HCPs.

**Figure S1. Anomalous signal of Fe atoms and reduced hybrid cluster architecture.**

**Figure S2. Residues conservation in structurally characterised HCPs.**

**Figure S3. Structural variation among HCP structures.**

**Figure S4. Electron density around the hybrid clusters in *Mt*HCP_mix_ structure.**

**Figure S5. Position of the Glu490 in the different HCPs structures.**

**Figure S6. Hydroxylamine reductase activity of *Mt*HCP.**

**Figure S7. Residues involved in the channelling system of *Mt*HCP.**

**Figure S8. 2-Methyl-pentane-2,4-diol obstructing the tunnel 2 in *Mt*HCP.**

**Figure S9. Channelling system in the different HCP structures.**

**Figure S10. Active site closure caused by oxidation in HCPs.**

**Figure S11. Phylogenetic tree of HCPs.**

**Table S1.** X-ray analysis statistics

|  | ***Mt*HCP_red_**  **SAD Fe K-edge** | ***Mt*HCP_red_** | ***Mt*HCP soaked with hydroxylamine** |
| --- | --- | --- | --- |
| **Data collection** |  |  |  |
| Synchrotron source | SLS, X06DA | SLS, X06DA | PETRAIII, P11 |
| Wavelength (Å) | 1.74012 | 1.23984 | 1.00004 |
| Space group | *P*2_1_2_1_2 | *P*2_1_2_1_2 | *P*2_1_2_1_2 |
| Resolution (Å) | 70.58 ̶ 1.72  (1.74 ̶ 1.72) | 51.06 ̶ 1.45  (1.48 ̶ 1.45) | 70.64 ̶ 1.36  (1.48 ̶ 1.36) |
| Cell dimensions |  |  |  |
| a, b, c (Å) | 97.46, 102.33, 58.84 | 97.56, 102.45, 58.90 | 97.77, 102.19, 58.32 |
| α, β, γ (°) | 90, 90, 90 | 90, 90, 90 | 90, 90, 90 |
| R_merge_(%)^a^ | 10.5 (59.8) | 11.9 (119.3) | 11.0 (106.1) |
| R_pim_ (%)^a^ | 4.5 (38.4) | 5.6 (64.2) | 4.3 (47.2) |
| CC_1/2_ ^a^ | 0.999 (0.811) | 0.996 (0.510) | 0.999 (0.611) |
| I/σ*_I_*^a^ | 17.6 (2.4) | 9.5 (1.4) | 11.5 (1.6) |
| Spherical completeness^a^ | 92.9 (82.0) | 98.8 (92.4) | 70.4 (15.8) |
| Ellipsoidal completeness^a^ | - | - | 94.9 (67.9) |
| Redundancy^a^ | 11.9 (5.6) | 5.3 (4.0) | 7.1 (5.9) |
| Nr. unique reflections^a^ | 59,213 (2,569) | 103,697 (4,833) | 89,372 (4,469) |
| Matthews coefficient *V*_M_ | 2.44 | 2.45 | 2.43 |
| Peptide chain in the AU | 1 | 1 | 1 |
| Solvent content (%) | 49.60 | 49.75 | 49.32 |
| **Refinement** |  |  |  |
| Resolution (Å) | - | 51.06 ̶ 1.45 | 51.09 ̶ 1.36 |
| Number of reflections | - | 103,571 | 89,365 |
| R_work_/R_free_^b^ (%) | - | 13.31/15.24 | 12.90/16.61 |
| Number of atoms |  |  |  |
| Protein | - | 4341 | 4393 |
| Ligands/ions | - | 68 | 160 |
| Solvent | - | 649 | 629 |
| Mean B-value (Å^2^) | - | 19.51 | 18.44 |
| Molprobity clash  score, all atoms | - | 1.47 | 1.21 |
| Ramachandran plot |  |  |  |
| Favoured regions (%) | - | 97.79 | 98.15 |
| Outlier regions (%) | - | 0 | 0 |
| r.m.s.d.^c^ bond lengths (Å) | - | 0.009 | 0.012 |
| r.m.s.d.^c^ bond angles (°) | - | 1.288 | 1.352 |
| **PDB ID code** |  | **8CNR** | **8CNS** |

^a^ Values relative to the highest resolution shell are within parentheses. ^b^ Rfree was calculated as the Rwork for 5 % of the reflections that were not included in the refinement. ^c^ R.m.s.d., root mean square deviation.

**Table S2.** Root mean square deviation (r.m.s.d.) and sequence identity between the *Mt*HCP structures and the structurally characterised HCPs. R.m.s.d. (and aligned Cα) after superposition using PyMOL or Secondary-Structure matching (from the CCP4 suite) are given in top and bottom line, respectively. *Dd:* *Desulfovibrio desulfuricans*, *Dv:* *Desulfovibrio vulgaris; Ec: Escherichia coli K-12; Mm: Methanothermobacter marburgensis str. Marburg*

|  | **R.m.s.d. (Cα) from *Mt*HCP_red_, PDB 8CNR** | **R.m.s.d. (Cα) from *Mt*HCP mixed state, PDB 8CNS** | **Sequence identity, % (coverage, %) to *Mt*HCP** |
| --- | --- | --- | --- |
| *Mt*HCP_red_, 8CNR | / | 0.137 (484) | 100(100) |
|  |  | 0.3182 (545) |  |
| *Mt*HCP_mix_, 8CNS | 0.137 (484) | / | 100 (100) |
|  | 0.3182 (545) |  |  |
| *Dv*HCP_red_, 1OA1 | 0.436 (459) | 0.435 (461) | 66.06 (98) |
|  | 0.7488 (525) | 0.7398 (527) |  |
| *Dv*HCP_ox_, 1GNT | 0.480 (456) | 0.449 (453) | 66.06 (98) |
|  | 0.7888 (525) | 0.7660 (527) |  |
| *Dv*HCP_ox_, 1E1D | 0.582 (473) | 0.565 (475) | 65.88 (98) |
|  | 0.8378 (525) | 0.7773 (525) |  |
| *Dv*HCP_ox_, 1E2U | 0.504 (465) | 0.477 (468) | 66.06 (98) |
|  | 0.7808 (525) | 0.7504 (527) |  |
| *Dv*HCP_ox_, 1W9M | 0.511 (466) | 0.477 (466) | 66.06 (98) |
|  | 0.7882 (525) | 0.7540 (527) |  |
| *Dd*HCP_red_, 1OA0 | 0.335 (428) | 0.377 (437) | 64.95 (98) |
|  | 0.7761 (534) | 0.7382 (532) |  |
| *Dd*HCP_ox_, 1GNL | 0.335 (428) | 0.336 (428) | 64.95 (98) |
|  | 0.7816 (534) | 0.7731 (535) |  |
| *Dd*HCP_ox_, 1GN9 | 0.374 (442) | 0.383 (443) | 64.95 (98) |
|  | 0.7886 (535) | 0.7404 (534) |  |
| *Dd*HCP_mix_, 1UPX | 0.339 (436) | 0.341 (436) | 64.95 (98) |
|  | 0.7739 (534) | 0.7620 (535) |  |
| *Ec*HCP_ox_, 7DE4 | 0.772 (395) | 0.785 (395) | 43.01 (98) |
|  | 1.3134 (509) | 1.3278 (510) |  |
| *Mm*HCP, 7E0L | 0.761 (325) | 0.762 (323) | 42.61 (98) |
|  | 1.0405 (493) | 1.1242 (500) |  |


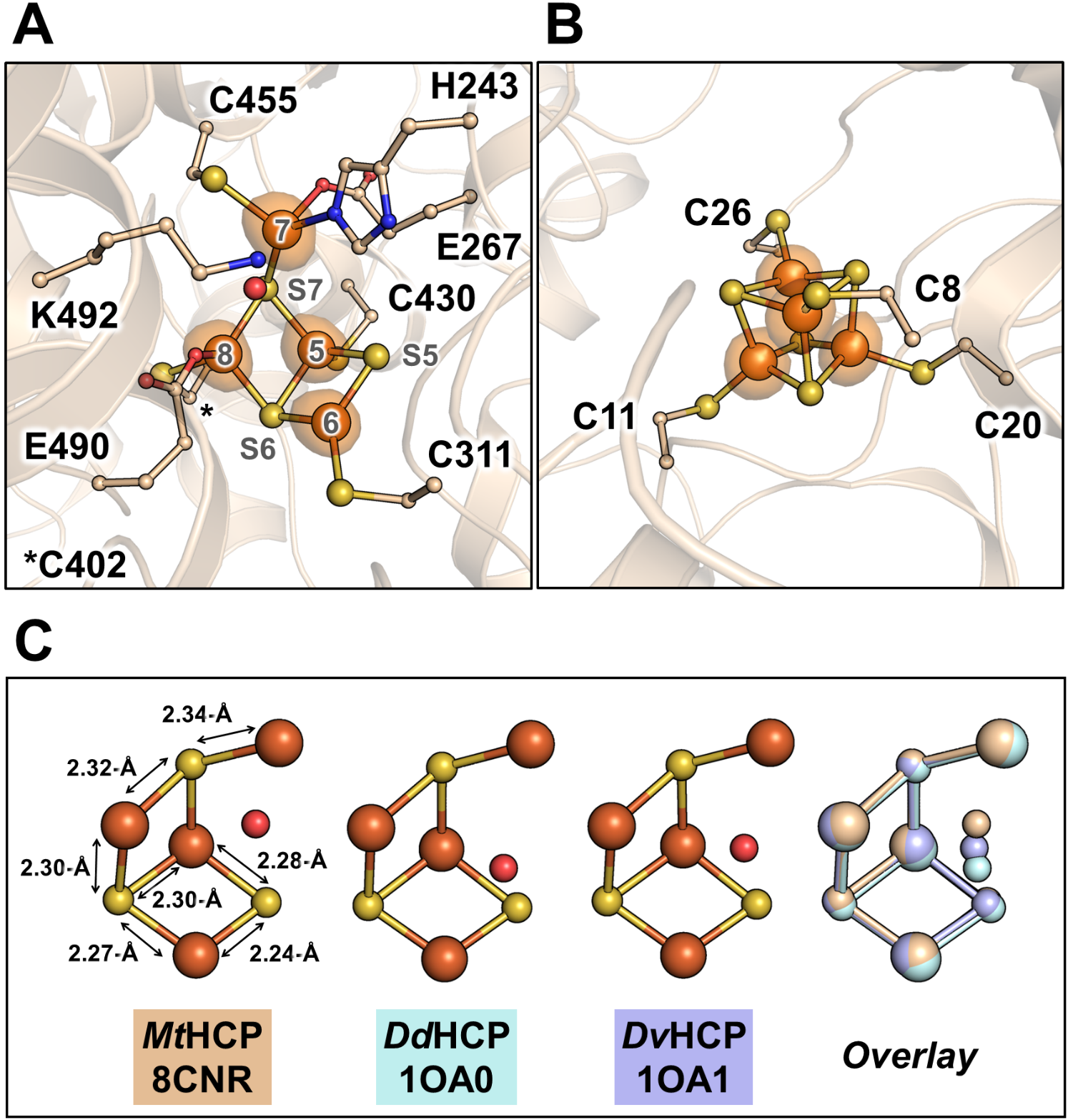


**Figure S1. Anomalous signal of Fe atoms and reduced hybrid cluster architecture. A, and B.** Structure of the *Mt*HCP in reduced state centred on the hybrid cluster (**A**) and the electron transfer [4Fe-4S] cluster (**B**). The anomalous map obtained from data collected at the Fe peak is displayed at a σ of 10 as a transparent orange surface. The peptidic chain is shown as cartoon coloured wheat. The clusters and the residues interacting with them are shown as balls and sticks. **C**. Structure of the hybrid cluster in the structures of the reduced *Mt*HCP, *Dd*HCP, *Dv*HCP and an overlay of the three. Clusters are shown as balls and sticks. The oxygen, nitrogen, sulphur and iron atoms are coloured red, blue, yellow and orange, respectively.

**
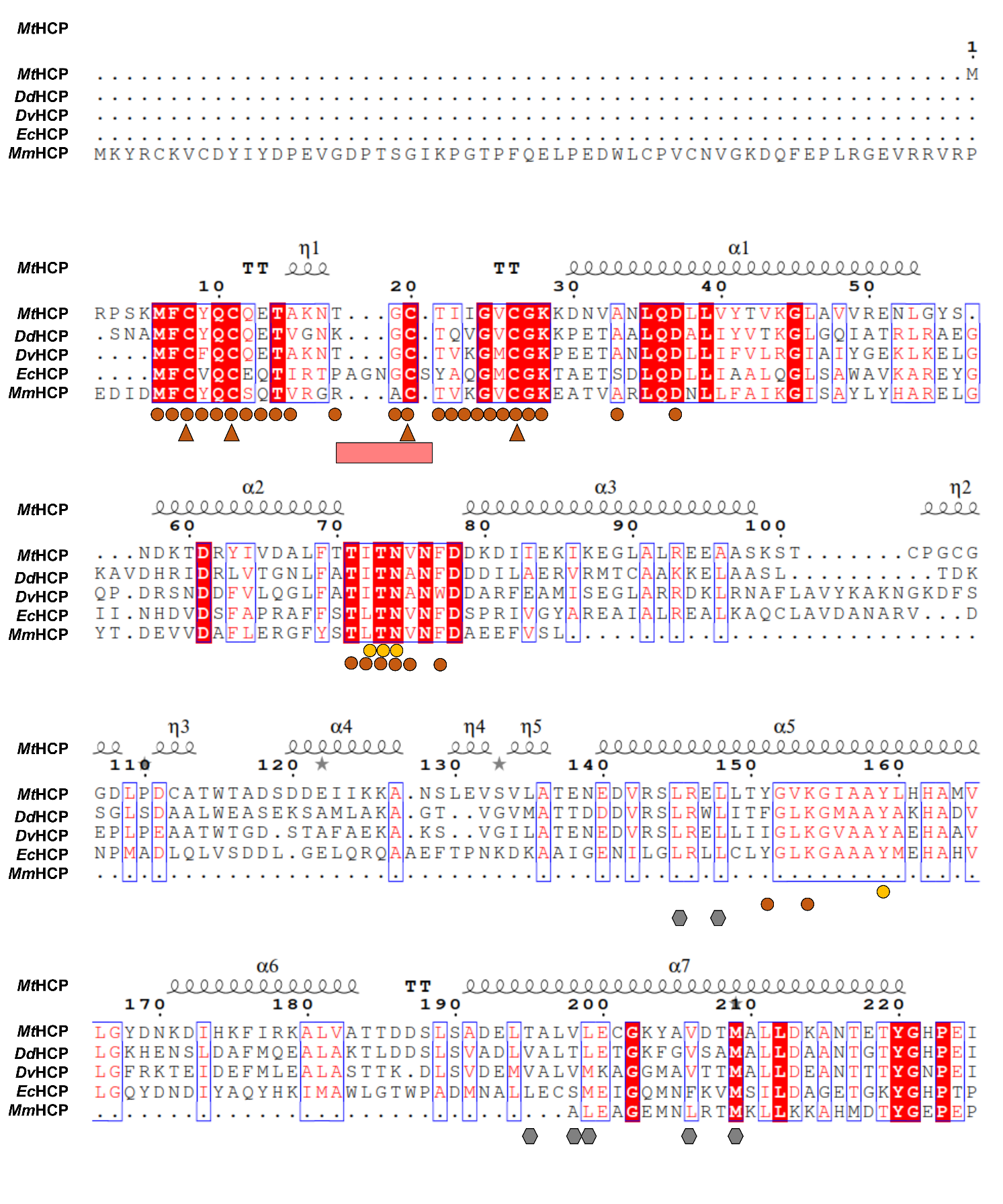

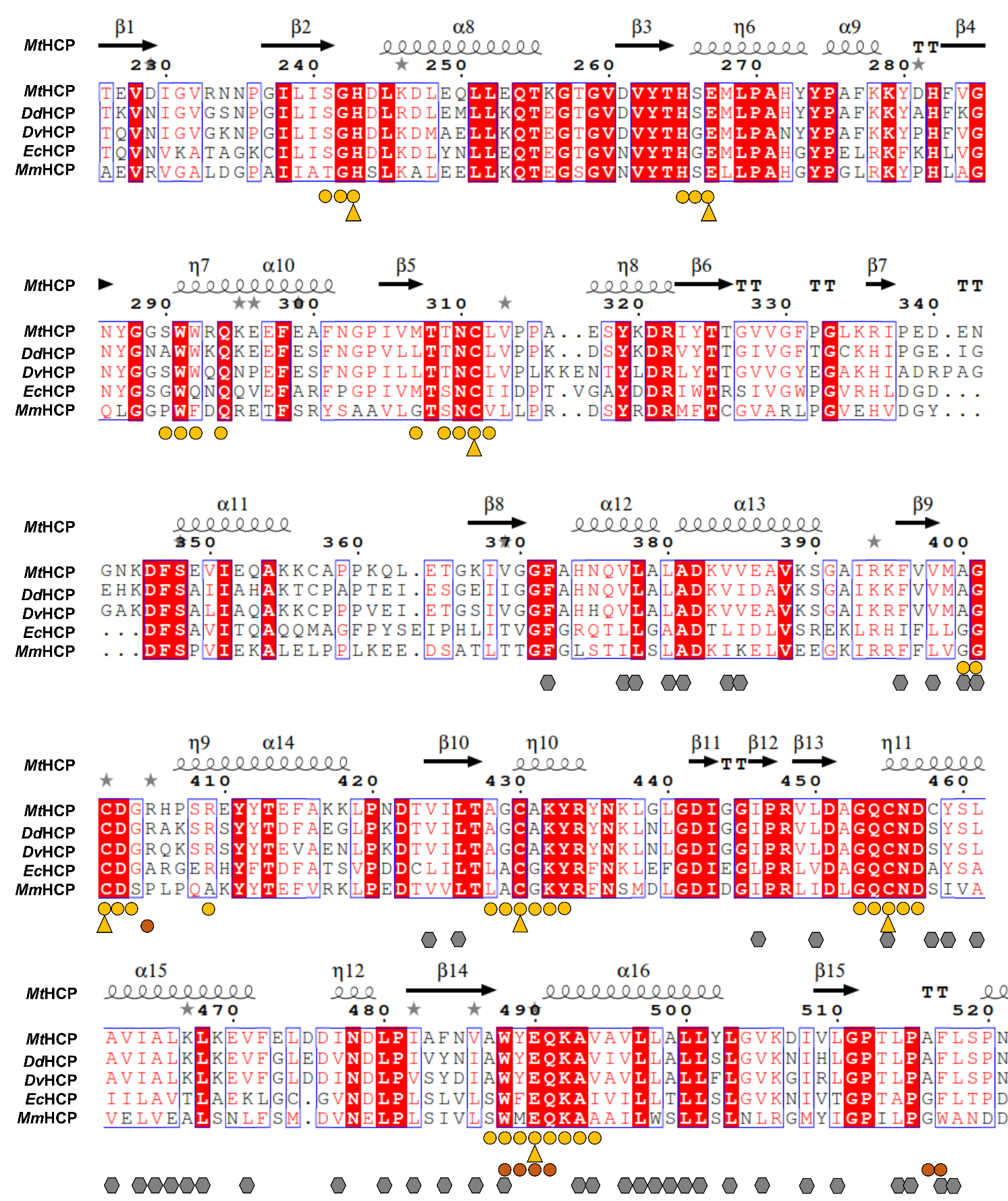

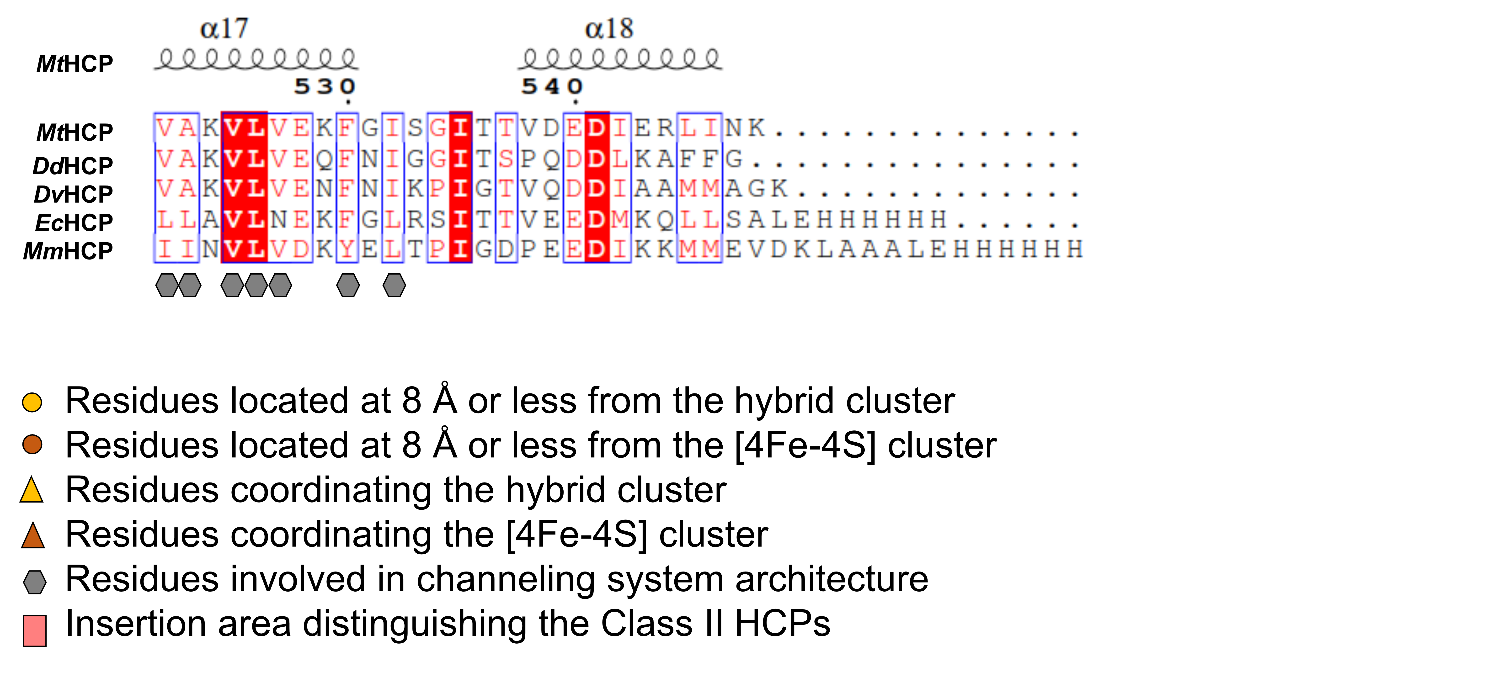
**

**Figure S2. Residues conservation in structurally characterised HCPs.** Sequence alignment of *Mt*HCP, *Dd*HCP, *Dv*HCP, *Ec*HCP and *Mm*HCP. Sequences were associated with the following PDB codes 8CNR (*Mt*HCPred), 1OA0 (*Dd*HCP), 1OA1 (*Dv*HCP), 7DE4 (*Ec*HCP) and 7E0L (*Mm*HCP). The alignments were designed by Espript on the structure of *Mt*HCP_red_. The residues from *Mt*HCP_red_ surrounding the clusters, coordinating the clusters and involved in tunnel architecture are highlighted. A red block highlights the insertion characteristic to Class II HCP. When applicable, the persulphido-cysteine (noted X in structure sequence) was replaced by a cysteine.


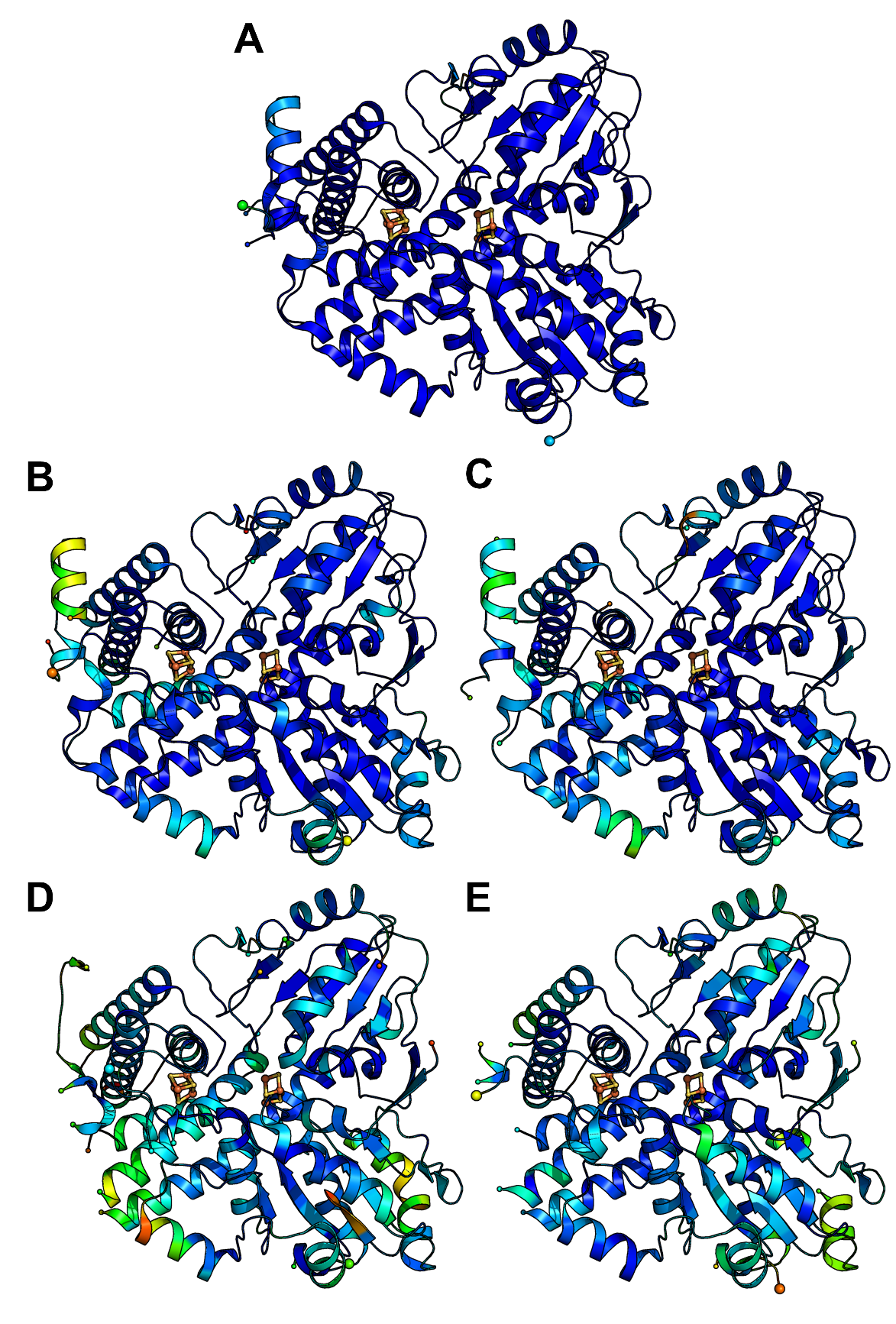


**Figure S3. Structural variation among HCP structures.** *Mt*HCP_red_ (PDB 8CNR) is represented as cartoon coloured by the residue deviation resulting from the superimposition with *Mt*HCP_mix_ (PDB 8CNS, **A**), *Dd*HCP_red_ (PDB 1OA0, **B**), *Dv*HCP_red_ (PDB 1OA1, **C**), *Ec*HCP (PDB 7DE4, **D**) and *Mm*HCP (PDB 7E0L, **E**). The colour range from blue to red (deviation ranging from 0.021 to 4.572 Å, respectively). Non-aligned residues, residues with aberrant deviation and fragments of three residues or less were omitted for clarity. Chain breaks are shown as spheres, with N- and C-termini exhibiting larger radii. The hybrid cluster and [4Fe-4S] cluster are shown as balls and sticks, with sulphur and iron being coloured yellow and orange, respectively.

**
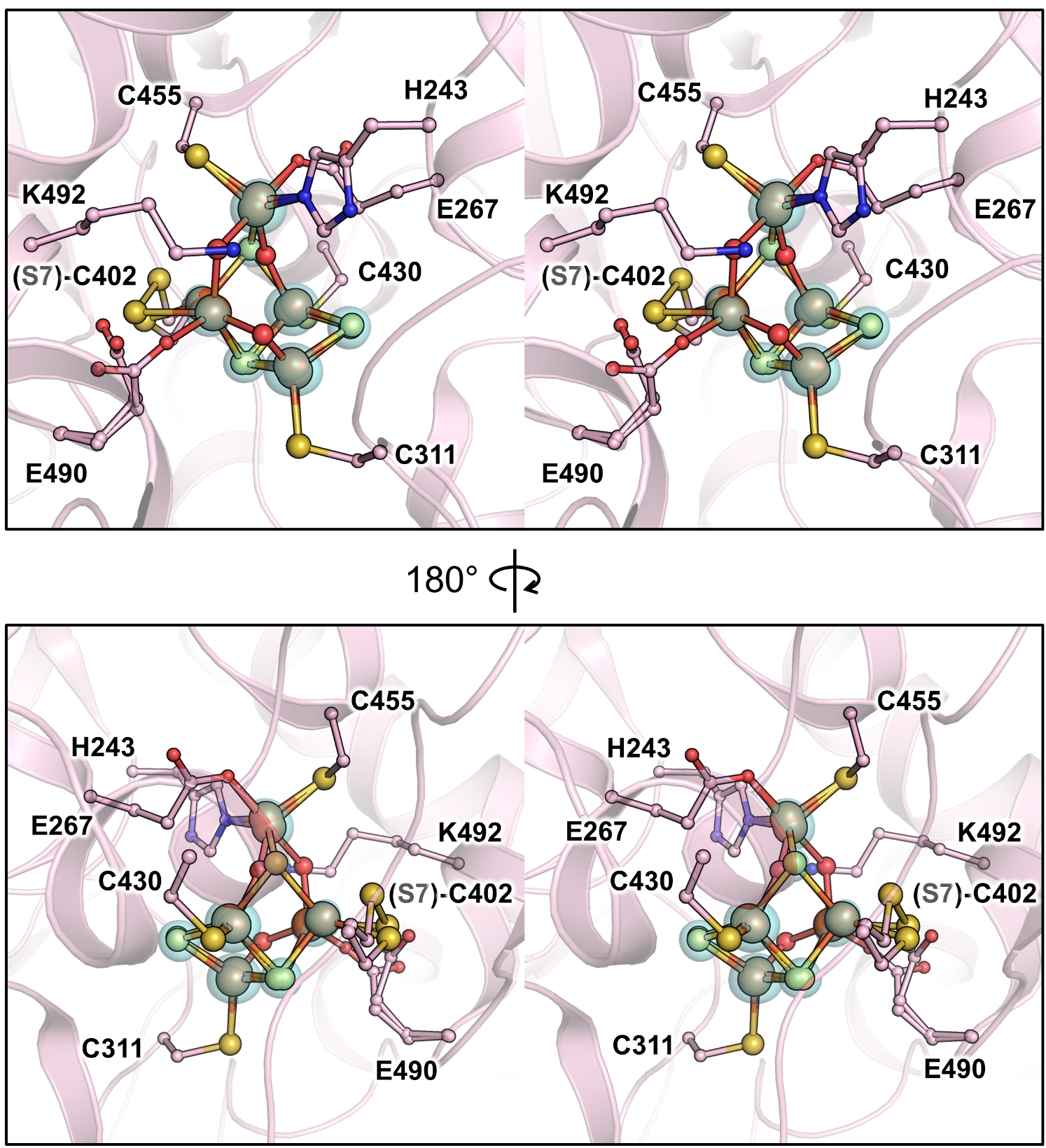
**

**Figure S4. Electron density around the hybrid clusters in *Mt*HCP_mix_ structure.** The stereo-view of the structure is shown in two orientations. The transparent cartoon is coloured in pink, with clusters and residues involved in coordination and interactions shown as balls and sticks. Oxygen, nitrogen, sulphur and iron atoms are coloured red, blue, yellow and orange, respectively. The electron density map (2*F*_o_*-F*_c_) is contoured only around Fe and S atoms (7 σ) from the cluster and coloured in an aquamarine blue surface.

**
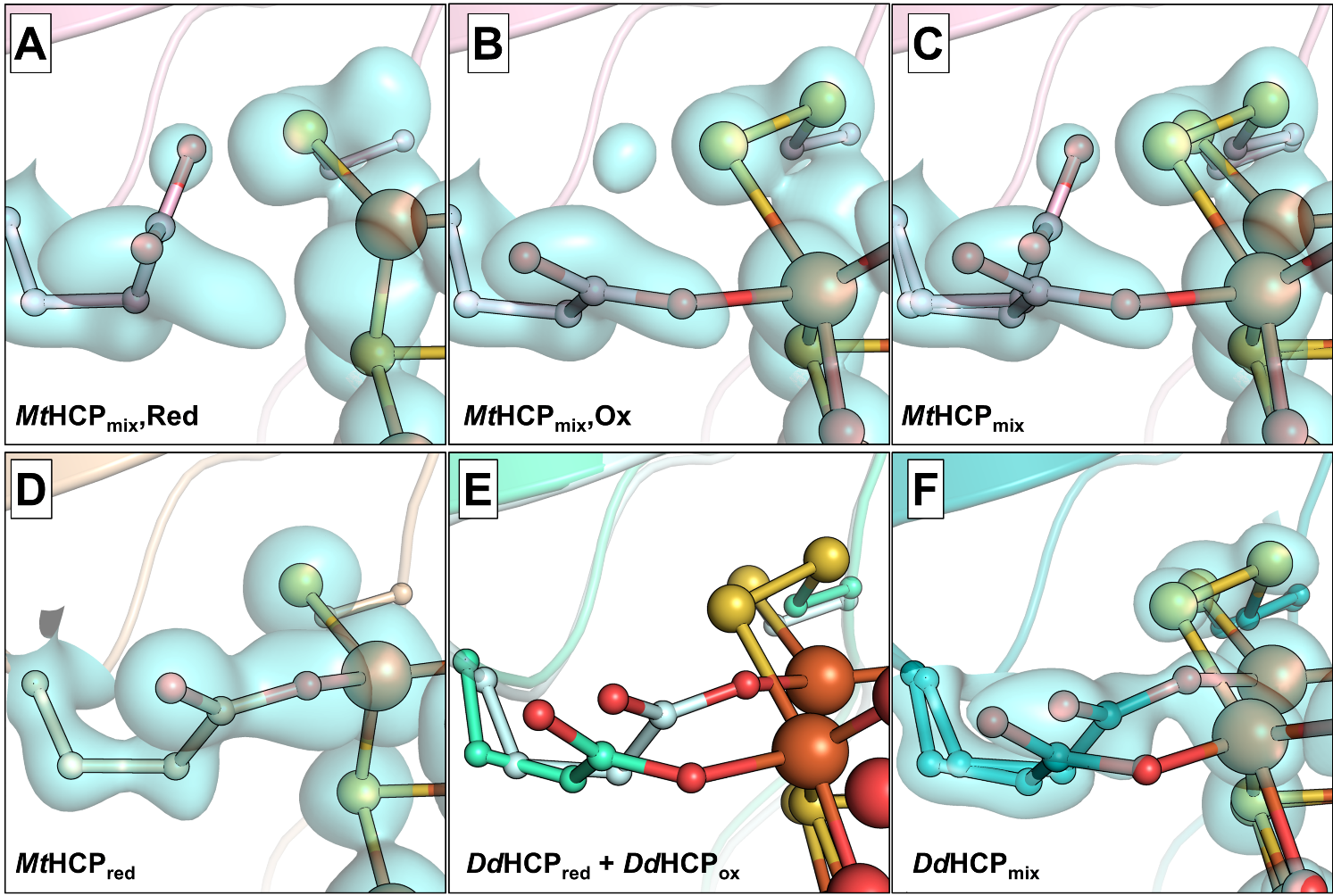
**

**Figure S5. Position of the Glu490 in the different HCPs structures. A-F.** Position of the glutamate 490 near the hybrid cluster in the structures of the reduced (**A**) and oxidised state (**B**) extracted from the *Mt*HCP_mix_ structure (**C**), the *Mt*HCP_red_ structure (**D**), the *Dd*HCP_red_ and *Dd*HCP_ox_ structures (1OA0 and 1GNL, **E**), and the *Dd*HCP_mix_ structure (1UPX, **F**). The electron density map (2*F*_o_-*F*_c_, contoured at 2 σ) is shown as an aquamarine blue transparent surface, when available. The structures are shown in transparent cartoons coloured wheat, light pink, light cyan, green cyan and aquamarine blue for the *Mt*HCR_red_, *Mt*HCP_mix_, *Dd*HCP_red_, *Dd*HCP_ox_ and *Dd*HCP_mix_, respectively. Clusters and residues coordinating or interacting with the hybrid cluster are shown in balls and sticks. Oxygen, nitrogen, sulphur and iron are coloured red, blue, yellow and orange, respectively.

**
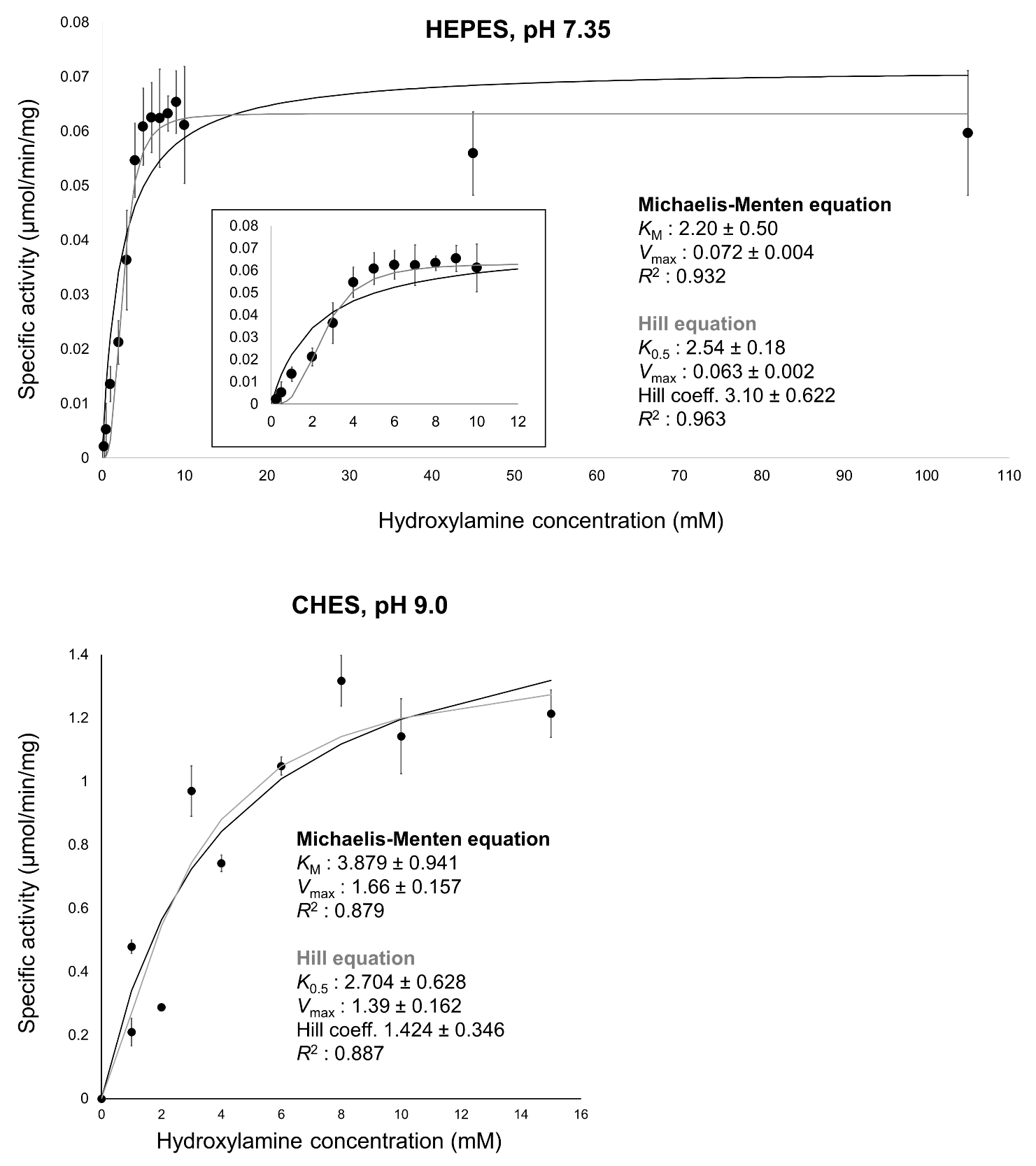
**

**Figure S6. Hydroxylamine reductase activity of *Mt*HCP.** The specific activity (in µmol of hydroxylamine reduced/min/mg of purified HCP) depending on the used substrate concentration in 50 mM HEPES, pH 7.35 (top) or 100 mM CHES, pH 9.0 (bottom) are shown as black dots. The *K*_M_, *K*_0.5_, *V*_max_ and the Hill coefficient obtained according to the Michaelis-Menten or Hill equation are indicated. The coefficient of determination (*R*^2^) obtained by comparing the raw data with the predicted values using the Michaelis-Menten (grey line) or the Hill equation (black line) is indicated. All measurements have been done in triplicates.

**
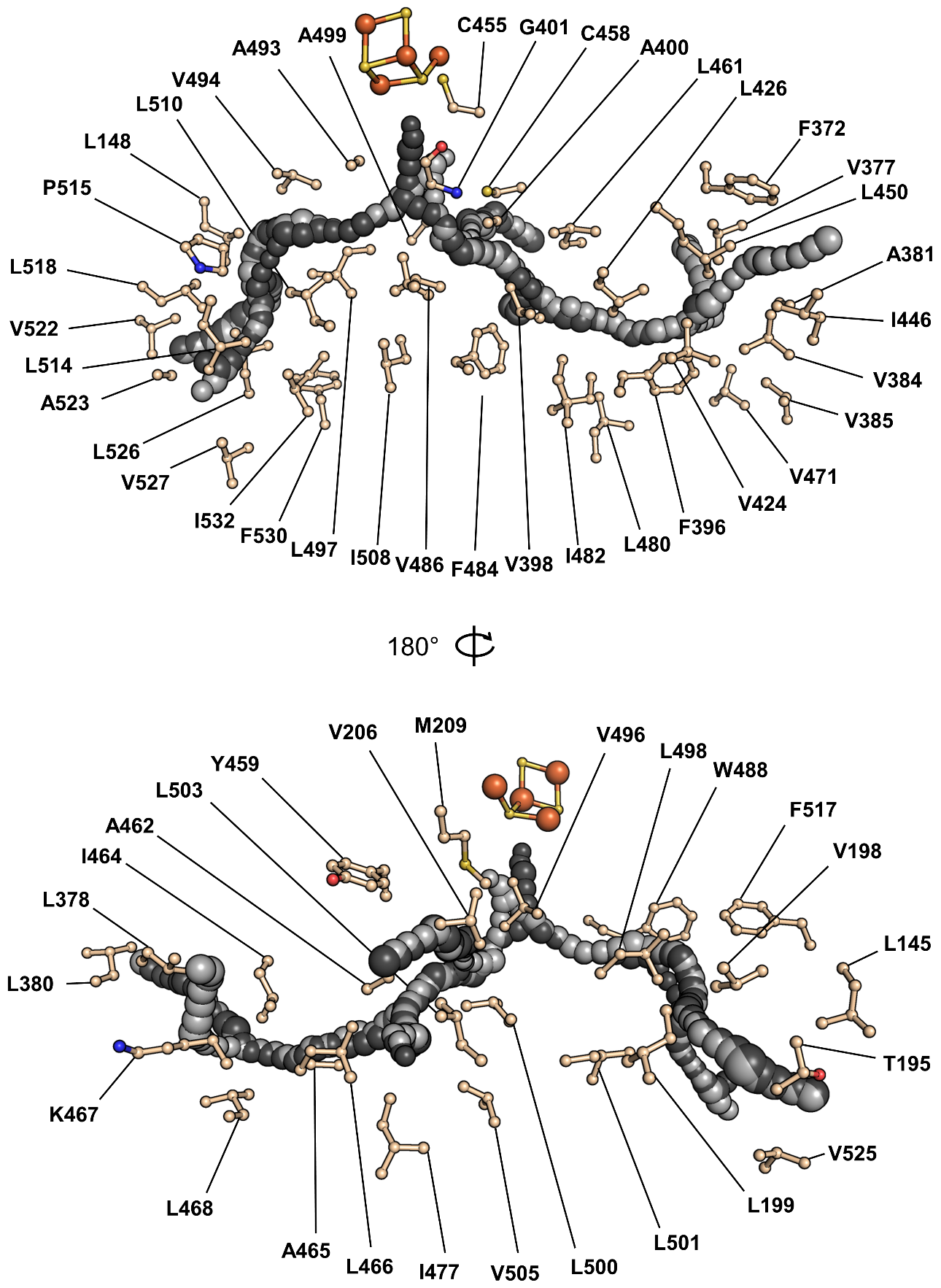
**

**Figure S7. Residues involved in the channelling system of *Mt*HCP.** The channelling system has been calculated by the *CAVER* program on *Mt*HCP_red_ (dark grey spheres) and *Mt*HCP_mix_ (light grey spheres). The residues contributing to the tunnels are shown as sticks and labelled. Only *Mt*HCP_red_ is represented for clarity. Carbon, oxygen, nitrogen and sulphur are coloured wheat, red, blue and yellow, respectively.

**
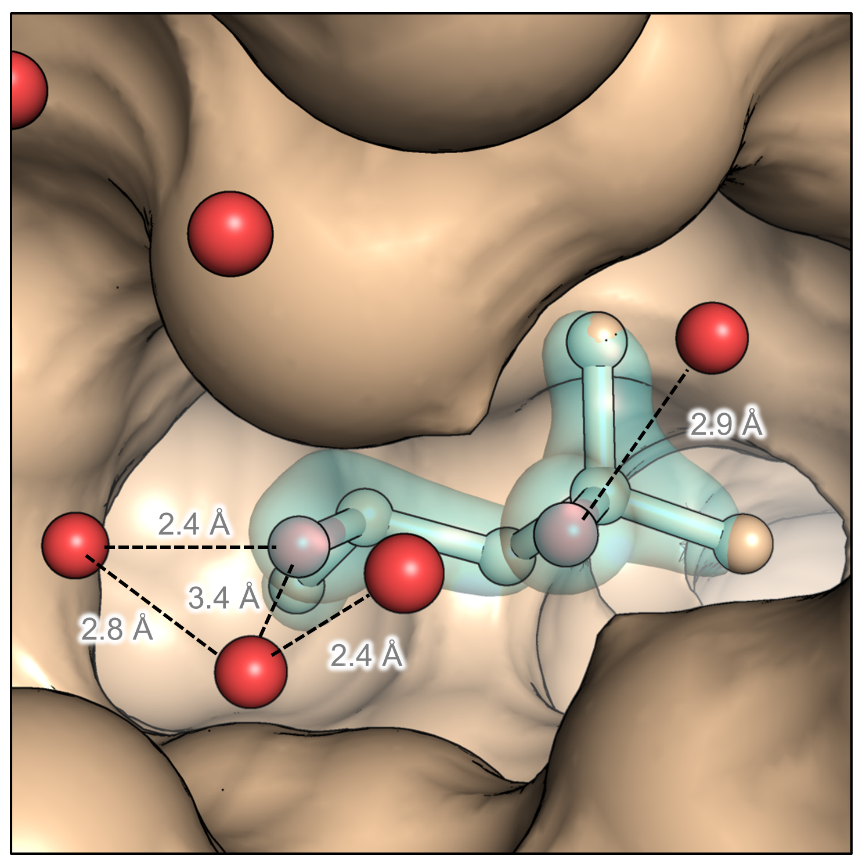
**

**Figure S8. 2-Methyl-pentane-2,4-diol obstructing the tunnel 2 in *Mt*HCP.** The molecule is located at the surface of the *Mt*HCP_red_ structure, coloured in wheat. A (4R)-2-methylpentane-2,4-diol molecule was modelled, according to the electron density (aquamarine blue surface, 2*F*_o_-*F*_c_, contoured at 1.5 σ). The residue is shown as balls and sticks and the interacting water molecules are shown as spheres. Oxygen is coloured red. Distances are indicated in Ångström and drawn as dashed black lines.

**
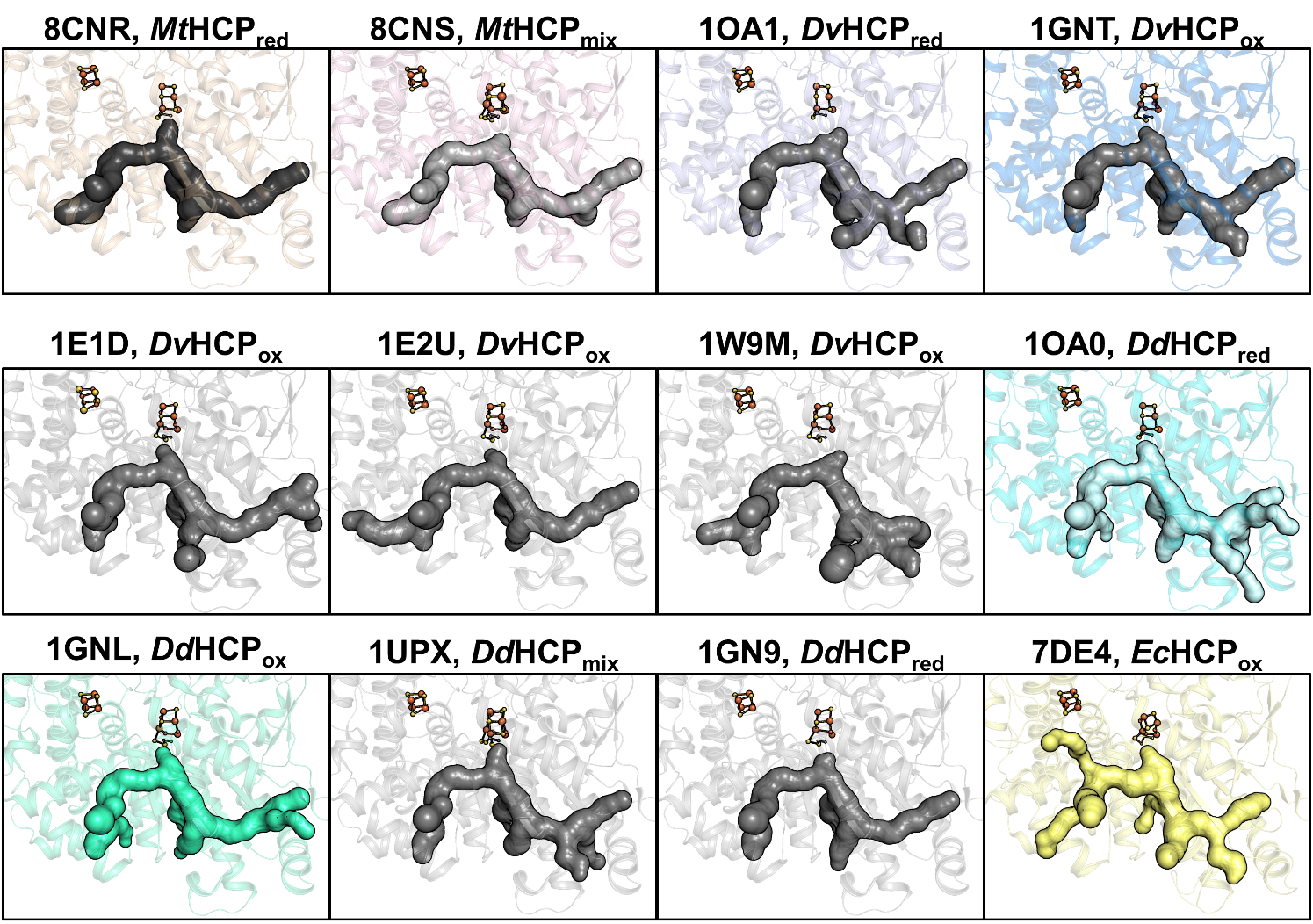
**

**Figure S9. Channelling system in the different HCP structures.** The different channelling systems predicted by the *CAVER* program on the HCP structures are shown as surface coloured grey or according to other figures (dark grey for *Mt*HCP_red_, light grey for *Mt*HCP_mix_, green cyan for *Dd*HCP_ox_, light cyan for *Dd*HCP_red_, yellow for *Ec*HCP_ox_). The proteins are shown in cartoon coloured grey or according to other figures (wheat for *Mt*HCP_red_, light pink for *Mt*HCP_mix_, light blue for *Dv*HCP_red_, marine blue for *Dv*HCP_ox_, pale cyan for *Dd*HCP_red_, green cyan for *Dd*HCP_ox_, light yellow for *Ec*HCP_ox_). The hybrid cluster and equivalent of cysteine/persulphido-cysteine 402 (*Mt*HCP numbering) are shown as balls and sticks, with sulphur and iron coloured yellow and orange, respectively.

**
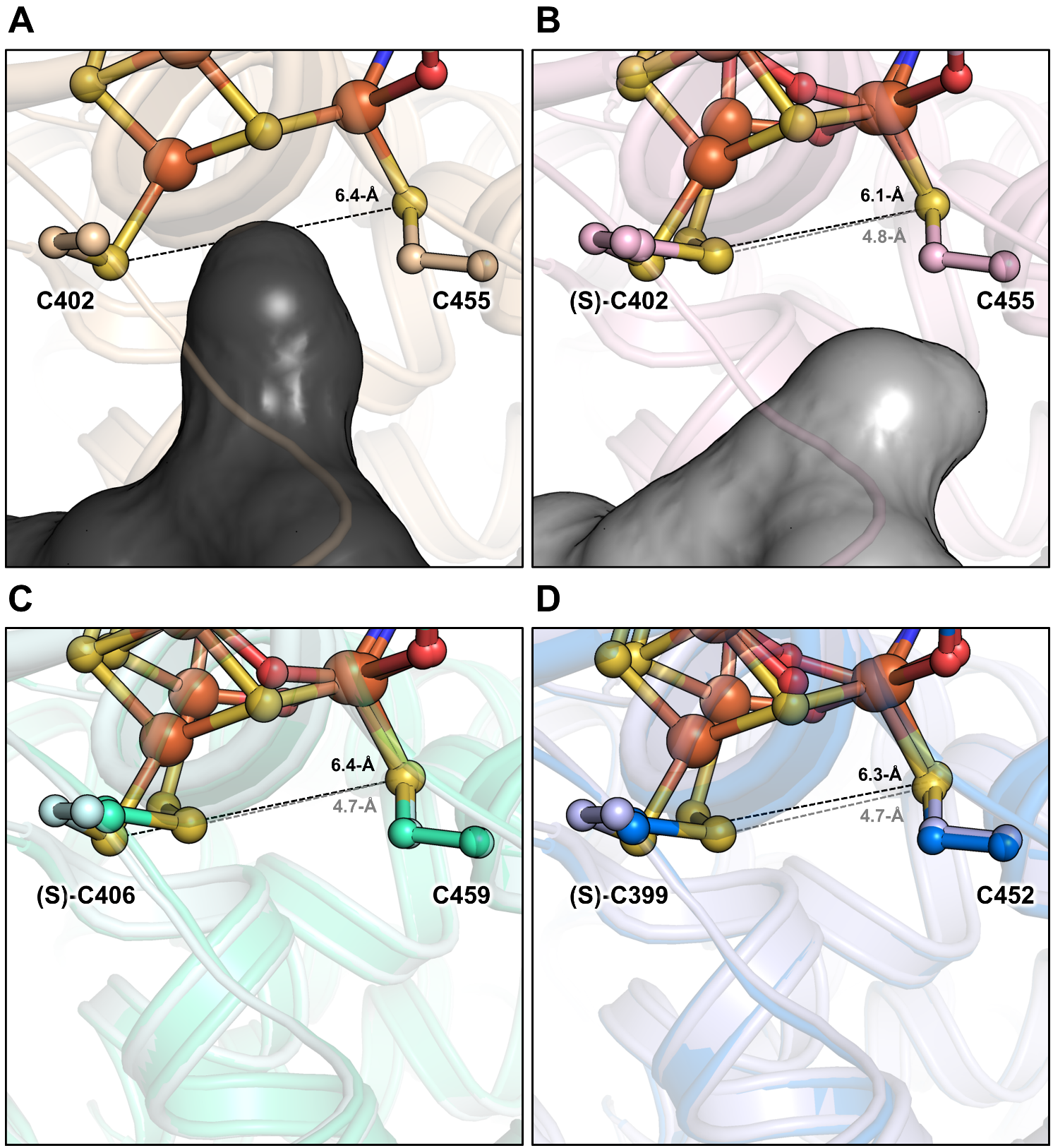
**

**Figure S10. Active site closure caused by oxidation in HCPs. A, B and C.** Active site accessibility in *Mt*HCP_red_ (**A**) and *Mt*HCP_mix_ (**B**). The distance between the distal sulphur of cysteine 402 and cysteine 455 is indicated. The channels predicted by *CAVER* on the structure of *Mt*HCP_red_ and *Mt*HCP_mix_ are represented as surface coloured in dark and light grey, respectively. **C, and D.** Superimposition of the structures of *Dd*HCP (reduced state PDB 1OA0, light cyan and oxidised state PDB 1GNL, green cyan) and *Dv*HCP (reduced state PDB 1OA1, light blue and oxidised state PDB 1GNT, marine blue). The distance between the distal sulphurs of cysteines in the reduced (black) and oxidised (grey).

**
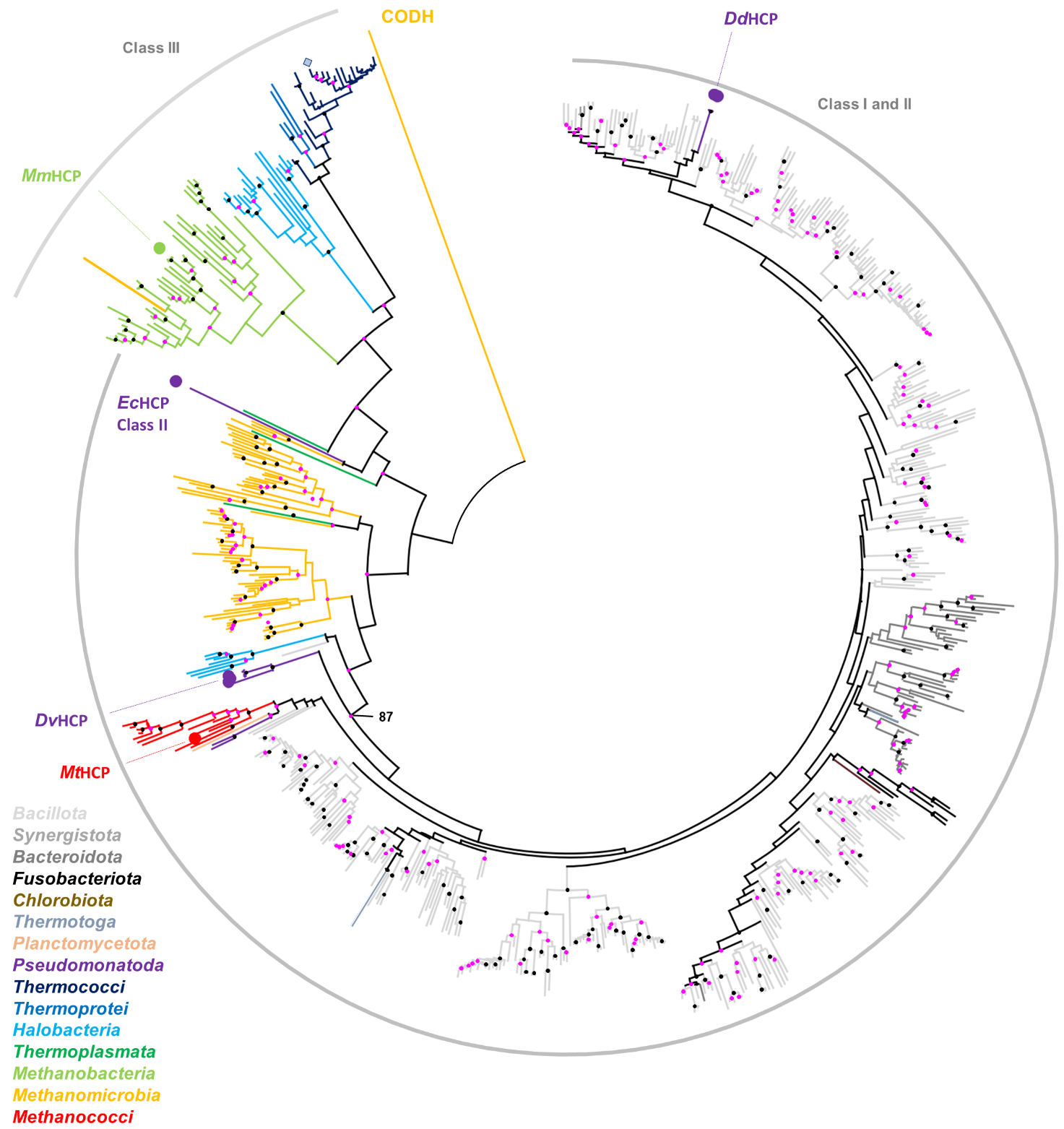
Figure S11. Phylogenetic tree of HCPs.** The tree was constructed with the maximum likelihood method from the sequences of structurally characterized HCPs, the 500 closest sequences to *Mt*HCP in the RefSeq database and the 500 closest sequences to *Mt*HCP in the same database but restricted to the *Archaea* domain. The duplicated sequences were manually deleted, as the sequence corresponding to CO-dehydrogenase only being kept as an outgroup. The monophylogenetic branches are coloured by phylum (bacteria) or class (archaea). Sequences from the PDB are highlighted by a dot and labelled. The sequence from *P. furiosus* is marked as a blue square. The CODH sequence used as an outgroup is labelled. The class III HCP and HCP I/II are indicated. Node statistics (200 replicates) between 50-90 and 90-100 are represented by pink and black dots, respectively.
